# Larval assemblages over the abyssal plain in the Pacific are highly diverse and spatially patchy

**DOI:** 10.7717/peerj.7691

**Published:** 2019-09-26

**Authors:** Oliver Kersten, Eric W. Vetter, Michelle J. Jungbluth, Craig R. Smith, Erica Goetze

**Affiliations:** 1 Hawaii Pacific University, Kaneohe, HI, United States of America; 2 Centre for Ecological and Evolutionary Synthesis (CEES), Department of Biosciences, University of Oslo, Oslo, Norway; 3 Department of Oceanography, University of Hawaii at Manoa, Honolulu, HI, United States of America

**Keywords:** Larval dispersal, Deep sea, Polymetallic nodule mining, Metabarcoding, Benthic Boundary Layer (BBL), Clarion Clipperton Fracture Zone (CCZ)

## Abstract

Abyssal plains are among the most biodiverse yet least explored marine ecosystems on our planet, and they are increasingly threatened by human impacts, including future deep seafloor mining. Recovery of abyssal populations from the impacts of polymetallic nodule mining will be partially determined by the availability and dispersal of pelagic larvae leading to benthic recolonization of disturbed areas of the seafloor. Here we use a tree-of-life (TOL) metabarcoding approach to investigate the species richness, diversity, and spatial variability of the larval assemblage at mesoscales across the abyssal seafloor in two mining-claim areas in the eastern Clarion Clipperton Fracture Zone (CCZ; abyssal Pacific). Our approach revealed a previously unknown taxonomic richness within the meroplankton assemblage, detecting larvae from 12 phyla, 23 Classes, 46 Orders, and 65 Families, including a number of taxa not previously reported at abyssal depths or within the Pacific Ocean. A novel suite of parasitic copepods and worms were sampled, from families that are known to associate with other benthic invertebrates or demersal fishes as hosts. Larval assemblages were patchily distributed at the mesoscale, with little similarity in OTUs detected among deployments even within the same 30 × 30 km study area. Our results provide baseline observations on larval diversity prior to polymetallic nodule mining in this region, and emphasize our overwhelming lack of knowledge regarding larvae of the benthic boundary layer in abyssal plain ecosystems.

## Introduction

More than 75% of the deep seafloor consists of abyssal plains that extend for thousands of kilometers between 3,000–6,000 m depths (e.g., Glover & Smith, 2003; Smith et al., 2008b; Vinogradova, 1997). Although punctuated by abyssal hills and seamounts (Harris et al., 2014; Durden et al., 2015), abyssal habitats are predominantly rolling plains covered by fine sediments, with some hard substrate in the form of polymetallic nodules and crusts (e.g., Glover & Smith, 2003; Hannides & Smith, 2003; Smith et al., 2008a). These ecosystems are characterized by low temperatures, very weak bottom currents, and low sediment accumulation rates, making them quiescent and physically relatively homogenous environments (e.g., Glover & Smith, 2003; Ramirez-Llodra et al., 2010; Hannides & Smith, 2003; Leitner et al., 2017). The vastness and remoteness of the abyssal plains makes them one of the largest and yet most poorly sampled ecosystems on our planet (Glover & Smith, 2003; Ramirez-Llodra et al., 2010).

Within the last few decades, there has been a substantial increase in the potential for anthropogenic impacts in the deep-sea (Smith et al., 2008b). Polymetallic nodules represent an extensive, although not yet exploited, mineral resource that has spurred interest in deep-sea mining, principally in the Clarion-Clipperton Fracture Zone (CCZ), a region in the equatorial North Pacific Ocean that contains high abundances of nodules rich in copper, nickel, cobalt, and rare earth elements (ISA, 2010; Hein et al., 2013). Sixteen exploration contracts, each covering up to 75,000 km^2^, have been granted by the International Seabed Authority (ISA) for polymetallic nodule mining within the CCZ (e.g., Wedding et al., 2013; Wedding et al., 2015). The CCZ is roughly 80% of the size of the contiguous United States (ca. 6,000,000 km^2^), and contains a variety of habitats, including abyssal plains, hills, seamounts, and fracture zones (e.g., Wedding et al., 2013; Wedding et al., 2015; Kaiser, Smith & Arbizu, 2017). Polymetallic nodules increase habitat heterogeneity in soft sediment regions, as they present hard substrate for microbial to megafaunal species (e.g.,  Amon et al., 2016; Shulse et al., 2017; Kaiser, Smith & Arbizu, 2017). Within the CCZ, variations in benthic community structure and function are primarily driven by north-south and east-west gradients in overlying productivity (Glover et al., 2002; Smith et al., 2008a; Wedding et al., 2013; Sweetman et al., 2018), and the diversity of meio—to macrofaunal invertebrates is high. Under-sampling and incomplete taxonomic information limit our understanding of abyssal biodiversity and biogeography (Smith et al., 2008a; McClain & Hardy, 2010), with many recent descriptions of new species and/or new distributional records for mega-, macro-, and meiofauna greatly increasing our knowledge of the CCZ fauna (e.g., Glover et al., 2016; Dahlgren et al., 2016; Wiklund et al., 2017; Goineau & Gooday, 2017; Taboada et al., 2018).

Many of the invertebrate macro- and megafaunal animals inhabiting the abyssal seafloor have a pelagic larval phase, enabling them to disperse on ocean currents, an essential component to the colonization of new habitats and resilience of deep-sea communities (e.g., McClain & Hardy, 2010; Mullineaux et al., 2010; Young et al., 2012). Following mining disturbance, pelagic larvae would serve as a primary vector for the re-establishment of seafloor communities (Wedding et al., 2013; Baco et al., 2016), and information about the abundance and spatio-temporal community structure of meroplankton in regions targeted for mining is therefore critical. Unfortunately, larval studies in the deep-sea remain challenging due to the remoteness of the habitat, low animal abundances, and difficulties in taxonomic identification due to the lack of taxonomic keys and a largely undescribed fauna (Adams, Arellano & Govenar, 2012; Kersten, Smith & Vetter, 2017). Several deep-sea studies address larval abundance and dispersal in bathyal hydrothermal vent systems and on mid-ocean ridges (e.g., Beaulieu et al., 2009; Mullineaux et al., 2005; Metaxas, 2011). However, there are almost no data on the flux or abundance of invertebrate larvae over abyssal plain habitats (Christiansen et al., 1999; Kersten, Smith & Vetter, 2017). Our prior work on these same samples using a traditional microscopy-based taxonomic approach describing the composition, abundance, and temporal variability of abyssal meroplankton provide the only prior larval data from the CCZ (Kersten, Smith & Vetter, 2017). Both larval abundances and fluxes in the CCZ were ∼1–2 orders of magnitude lower than those observed in mid-ocean ridge and hydrothermal-vent habitats (Kersten, Smith & Vetter, 2017), and meroplankton occurred almost exclusively within the Benthic Boundary Layer (BBL; sampled at 11 m above bottom (mab); Kersten, Smith & Vetter, 2017), which could have significant implications for the influence of near-bottom sediment plumes created by mining on the dispersal and recruitment of benthic populations.

For the first time, we use metabarcoding to investigate the diversity of a larval assemblage in the deep sea. Metabarcoding has routinely been used to characterize the diversity and structure of microbial communities (e.g., Sogin et al., 2006; Huber et al., 2007), with more recent applications to communities of benthic meiofauna and macrofauna (e.g., Fonseca et al., 2010; Leray & Knowlton, 2015), upper ocean zooplankton assemblages (e.g., Lindeque et al., 2013; Sommer et al., 2017; Hirai et al., 2017; Djurhuus et al., 2018), as well as gut contents and sediment communities (e.g., Leray et al., 2013; Deagle et al., 2013; Sinniger et al., 2016). Molecular methods circumvent some of the particular challenges of microscopy-based investigations of deep-sea meroplankton, including the common occurrence of damaged specimens and near ubiquitous lack of identification guides. The metabarcoding approach relies on reference databases that link morphological and molecular information for a wide diversity of taxa, and building and maintaining such resources for the deep sea is important to increasing the classification power of metabarcoding methods (e.g., Glover et al., 2015; Sinniger et al., 2016; Glover et al., 2018). Yet even in the absence of complete reference databases, metabarcoding methods capture sequences found in the CCZ, providing valuable baseline data on organismal occurrence and community diversity, as well as facilitating the inference of biogeographic distributions for species that have not yet been formally described.

**Figure 1:**
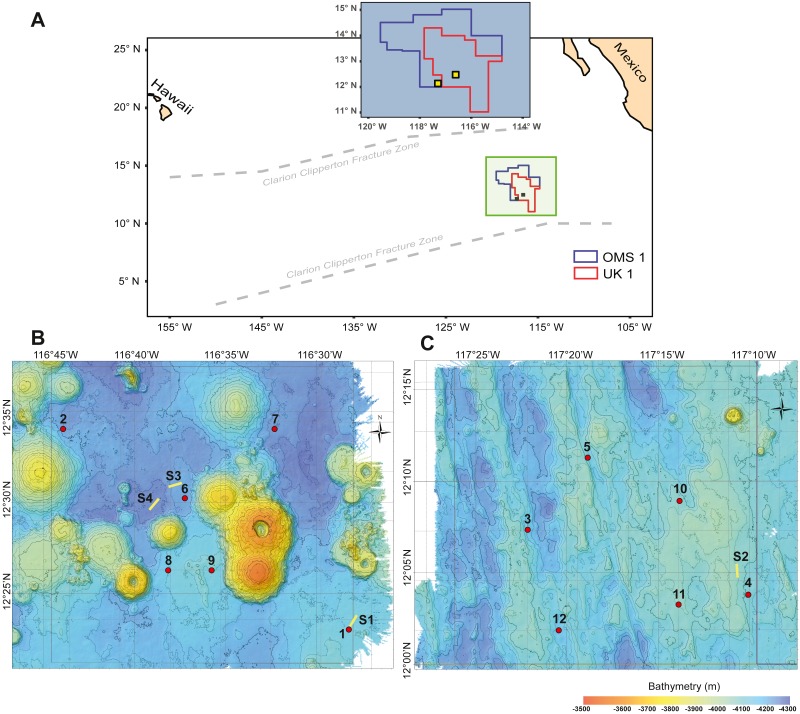
Study area with deployment locations. (A) Schematic map of the study areas within the OMS1 and UK1 mining claim areas in the Clarion Clipperton Fracture Zone. Small yellow squares represent the 30 × 30 km survey areas in the OMS01 and UK01 strata (UK1 stratum B and OMS1 stratum A). (B) Bathymetric map of UK1 Stratum B. (C) Bathymetric map of OMS1 Stratum A. In both B and C, red dots mark the abyssal plankton pump deployment sites; yellow lines show the epipelagic plankton tows.

This study focuses on the meroplanktonic larval stages of benthic animals sampled over the near-bottom abyssal plain within two mining exploration contract areas in the Eastern CCZ (Fig. 1). Our goals are to assess species richness, diversity, and spatial variability of the larval assemblage at the mesoscale, and to evaluate the suitability of the metabarcoding approach to describe a deep-sea, near-bottom meroplankton assemblage through comparison to conventional taxonomic analyses of quantitative splits of the same samples (Kersten, Smith & Vetter, 2017). Demersal plankton samples were collected with autonomous plankton pumps above the seafloor, and the fauna characterized using three markers in the nuclear 18S rRNA and mitochondrial cytochrome *c* oxidase subunit I (mtCOI) genes. Our results provide important baseline records of larval diversity over the severely under-sampled abyssal plain ecosystem of the CCZ.

## Materials and Methods

### Plankton sample collections

During ABYSSLINE research cruise AB02, plankton samples were collected at six randomly chosen sites within each of two 30 × 30 km study areas, UK01 (United Kingdom 01) Stratum B and OMS01 (Ocean Minerals Singapore 01) Stratum A (Fig. 1). The center points of the two strata were approximately 100 km apart, and distances between stations within each study area ranged from 11–35 km. Study sites were sampled with two plankton pumps (McLane Large Volume Water Transfer System WTS-LV30; McLane Research Laboratories) mounted on a free-vehicle, yielding one sample per pump at each site. One of the pumps was irreparably damaged after the first two deployments, and the remaining ten stations were sampled with a single pump. Plankton were collected on 63-µm Nitex mesh filters over a 23-hr pumping period at about three m above the seafloor (Table S1). During each deployment, a waiting period of a minimum of 90 min was implemented between arrival of the free vehicle on the seafloor and starting of the pumps in order to allow for the settlement of resuspended sediment due to the landing of the sampling equipment. A positively buoyant plastic ball ensured sealing of the pump inlet during inactivity and return to the surface to prevent zooplankton contamination from non-target depths. To assess any unintentional zooplankton capture, oblique epipelagic zooplankton tows were conducted during both night and day at three stations in the UK01 stratum and one station in the OMS01 stratum (Fig. 1).

Upon shipboard recovery, plankton pump filter holders were quickly removed from the free-vehicle and disassembled in a cold lab (4 °C). Filters were transferred to chilled, GF/F-filtered seawater and sample material was gently washed off the filters. Zooplankton was quantitatively split using a Folsom plankton splitter, with one half fixed in 95% ethanol for molecular analyses and the other in 4% buffered formaldehyde for morphological studies. The ethanol-preserved samples were stored at 4 °C for 12–24 hrs, followed by an exchange of ethanol and subsequent storage at −20 °C. Prior to DNA extraction in the laboratory, larvae identifiable as pre-adult stages of benthic species were sorted from the ethanol-preserved fraction and removed for individual taxonomic identification and DNA barcoding. Additional details about sample collection are reported in File S1.

### Metabarcoding: DNA extraction, polymerase chain reaction (PCR), and sequencing

Prior to DNA extraction, excess ethanol was removed from each sample, and filters were retained through the initial overnight lysis. DNA was extracted using the E.Z.N.A. HP Tissue DNA Maxi kit (OMEGA), following the manufacturer’s protocol, and was eluted 3X, using the elution with the highest concentration of high molecular weight DNA for subsequent amplification and sequencing. Two regions of the nuclear 18S rRNA gene spanning the variable regions V1&V2 (∼365 bp fragment) (Fonseca et al., 2010) and V7&V8 (∼325 bp fragment) (Machida & Knowlton, 2012), and a ∼313 bp fragment of the mitochondrial cytochrome *c* oxidase subunit I (mtCOI) gene (Leray et al., 2013) were amplified by polymerase chain reaction (PCR) (see Table S2 for details). 18S rRNA and mtCOI were chosen to provide taxonomic resolution across the full assemblage (18S) and as close to the species level as possible (mtCOI), with primers selected to amplify across a wide range of taxonomic groups as shown in previous studies (e.g., Lindeque et al., 2013; Deagle et al., 2018). However, the exact taxonomic biases of these primers are not known (Fonseca et al., 2010; Machida & Knowlton, 2012; Leray et al., 2013), and we refrain from drawing any substantial ecological conclusions based on inter-marker comparisons. PCR products from triplicate reactions were combined, quantified using the Qubit dsDNA Broad-Range Assay Kit (Life Technologies), normalized across markers, and pooled into a single PCR template per sample for library preparation. Libraries were created using the TruSeq Nano kit (Illumina), with size selection targeting a 550-bp insert. Prior to sequencing, library fragment length and concentration were measured using the Agilent 2100 Bioanalyzer and quantitative PCR, respectively (Evolutionary Genetics Core Facility, Hawaii Institute of Marine Biology). One library for each of 18 samples (four epipelagic, 14 abyssal—two pumps at first two sites) was sequenced on the Illumina MiSeq system using V3 chemistry (300-bp, paired-end). Additional methodological details are reported in File S1.

### Metabarcoding: bioinformatics and classification

Demultiplexed, trimmed and paired reads were merged in PEAR (v 0.9.6, Zhang et al., 2014), and subsequent steps were performed in mothur (v1.38.0; Schloss et al., 2009), with guidelines as outlined in the MiSeq SOP (Schloss et al., 2009; Kozich et al., 2013). In mothur, sequences were filtered with the following parameters: no ambiguous bases, maximum homopolymer length of 10 bp, and sequence length between 295–355 bp (18S_V7&8), 335-395 bp (18S_V1&2) and 279–339 bp (mtCOI). Unique 18S sequences were aligned to the SILVA128 database (Quast et al., 2013; Yilmaz et al., 2014) and trimmed to remove sequences that aligned outside the target region. For the mtCOI amplicon, sequences were aligned to a custom reference database (modified and aligned version of the MIDORI_Longest database from Machida et al., 2017) using Multiple Alignment of Coding Sequences (MACSE) (Ranwez et al., 2011), and trimmed to remove sequences that aligned outside the target region. Duplicate sequences of all three markers were removed, and unique sequences were pre-clustered at 99% (18S) and 97% (mtCOI) similarity prior to identifying and removing chimeras using VSEARCH (Rognes et al., 2016).

Taxonomy was assigned to non-chimeric sequences based on the eukaryotic portion of the Silva128 database (18S), as well as the dereplicated MIDORI_Longest database (Machida et al., 2017; mtCOI), using a naïve Bayesian classifier (Wang method) with taxonomic levels at <80% bootstrap support discarded (Wang et al., 2007). Sequences assigned taxonomy within Holozoa were retained for further analyses. The remaining sequences for all three markers were clustered into OTUs at 99% (18S) and 97% (mtCOI) similarity using the average neighbor method, and consensus taxonomy for each OTU was assigned. Clustering the sequences into OTUs was the preferred analysis choice to implement a comparable analysis workflow across all markers. While amplicon sequence variants (ASVs) may be appropriate for the 18S data, they represent mtCOI haplotypes, or intraspecific genetic variation and would prevent a desired putative species-level analysis. OTUs that were present in both abyssal and epipelagic samples were discarded, with OTUs present only in abyssal samples retained for further analyses. In order to avoid inclusion of spurious low read count OTUs, OTUs with a relative abundance <0.01% across all samples were removed for each marker. The most common sequence within each OTU is hereinafter referred to as the representative sequence. For 18S OTUs, both the Silva taxonomy and blastn sequence similarity of the representative sequence were used to classify OTUs. Species names were reported for sequences with ≥99% sequence identity to the top hit in NCBI. In the absence of ≥99% sequence identity to any sequences in NCBI, ≥97% and ≥90% sequence similarity cutoffs, as well as the SILVA taxonomy, were used to assign OTUs to a family or order level, respectively. For mtCOI OTUs, we additionally performed BLASTx searches of OTU representative sequences against the NCBI nt database. For mtCOI, a species name was assigned to an OTU if the top hit was within a) 97% (blastn) or b) 99% (blastx) sequence similarity. If these cutoffs were not reached, ≥90% and ≥85% sequence similarity to the top blastn hit, as well as the SILVA taxonomy, were used to assign OTUs to a family or order level, respectively. In the absence of a clear differentiation between holo- and meroplankton due to a lack of taxonomic resolution, a phylogenetic approach implemented in the Statistical Assignment Package (SAP) (Munch et al., 2008) was also used. Taxonomic inferences for each OTU therefore include consideration of the SILVA classification, the % identity to NCBI reference sequences and the SAP assignment (where applied). Additional details on bioinformatic methods are reported in File S1. An overview of publicly available sequence and metadata files including file name, content, accession numbers and DOI’s are presented in Table S3. FASTQ files are available under the SRA accession numbers SRR9304913, SRR9304914, and SRR9304915. Metadata and OTU tables are available under the DOI 10.5061/dryad.vb68g9d.

### Metabarcoding: community analyses

Using the R (R Core Team, 2018) package *vegan* (Oksanen et al., 2018), rarefaction curves for each marker were calculated by stratum and for the entire eastern CCZ using 100 runs to assess both sequence coverage and zooplankton community diversity. Prior to community analyses, all stations were randomly subsampled (100X) to the lowest number of reads per station for each marker in order to correct for sequencing bias (Kozich et al., 2013). The median read count for each OTU at each station was used for further community analyses, and Good’s coverage index (Good, 1953) was calculated to assess sequence coverage at all stations post subsampling. To investigate coverage of the meroplankton richness in the two strata, species accumulation, Bootstrap, Jackknife1 and Chao1 curves were estimated with *vegan* using 100 permutations (Gotelli & Colwell, 2001; Magurran, 2004).

In order to facilitate comparisons between morphological and molecular analyses of these samples (Kersten, Smith & Vetter, 2017), only meroplankton OTUs were retained and analyzed in downstream community analyses. Meroplankton OTUs were defined as invertebrate taxa that are restricted in distribution to the benthos (epibenthic, infaunal) and/or that are parasitic on other invertebrates or vertebrates. Fish OTUs were included. Taxa that are commonly sampled using plankton nets, including several typical members of the BBL, such as the bathypelagic/benthopelagic calanoid family Phaennidae or the polychaete genus *Swima*, were excluded. Taxa with both pelagic and benthic representatives, such as Ostracoda or Isopoda, that lacked sufficient taxonomic resolution to classify them as exclusively benthic or parasitic were not included. Results from holoplanktonic OTUs, including BBL taxa, will be reported on separately.

Taxonomic overlap in meroplankton diversity recovered by the three metabarcoding markers was visualized by Venn diagrams created using InteractiVenn (Heberle et al., 2015) and phylograms inferred with phyloT (Letunic, 2018) and iTOL v3 (Letunic & Bork, 2016). The Shannon–Weaver index (H′, Shannon & Weaver, 1948), Simpson diversity index (Simpson, 1949), and Pielou’s evenness (Pielou, 1966) were calculated for each station in order to investigate differences in meroplankton diversity and evenness between the two strata. Differences in richness were assessed by Jacknife (first-order) and Bootstrap methods (Magurran, 2004). Similarity of composition and structure of the meroplankton assemblage between samples was evaluated using a hierarchical cluster analysis (group-average linking) based on the Bray–Curtis (Sorensen) similarity index and non-metric multidimensional scaling (NMDS) ordination (Kruskal & Wish, 1978), using a presence-absence transformation. To test for significant differences between the *a priori* defined groups (UK vs. OMS), ANOSIM (Clarke, 1993), PERMANOVA using distance matrices (‘*adonis’* function in R, Anderson, 2001), and Multi Response Permutation Procedure (MRPP, Mielke, Berry & Johnson, 1976) analyses were conducted. Multivariate homogeneity of groups dispersions was tested to assess the validity of the ANOSIM tests (Anderson, 2006). A Mantel test (Mantel, 1967) using Euclidean distances between stations was performed to test for a relationship between geographic distance and ecological distance in NMDS ordination space between stations.

### DNA barcoding of individual larvae

DNA extractions of sorted specimens of larval gastropods and bivalves used the whole animal, with the shell crushed during extraction. Larval polychaetes often retained debris on their chaetae or parapodia, and were cleaned prior to extraction. Most polychaete specimens <1.0 mm were extracted in their entirety. For larger polychaetes (>1.0 mm, juvenile dispersal stage), an anterior portion was saved as a voucher for identification by morphology, and the posterior portion of the body was used for DNA extraction. Genomic DNA was extracted using the DNeasy Blood & Tissue Kit (Qiagen), following manufacturer’s protocols. Subsequently, fragments of 18S (∼1,800 bp) and mtCOI (∼650 bp) were amplified by PCR (Table S2), using the conditions described above and in File S1. Sequences from both strands were aligned, checked for sequencing errors, and trimmed to match the regions from the metabarcoding primer sets to obtain a consensus sequence (Geneious v9.1.5). Sequences from each marker were classified using the same approach as for the metabarcoding OTU representative sequences (SILVA, blastn, SAP) to obtain a consensus taxonomy. Sequences were also blasted against a local reference database composed of representative sequences from metabarcoding OTUs in order to compare the barcoding and metabarcoding dataset. Additional details on the barcoding methods are found in File S1.

## Results

### Metabarcoding—bioinformatics and sequence coverage

Over 6.9 million sequences were obtained for the 18S_V1&2, 18S_V7&8, and mtCOI amplicons combined. After demultiplexing and quality filtering, 92.7–99.7% of reads were retained, of which 24–34% were unique. Downstream processing steps within mothur resulted in the loss of 5–53% of sequences (chimera removal, exclusion of non-metazoan reads), and the remaining sequences were clustered into 9,793–163,661 OTUs (99% similarity 18S, 97% similarity mtCOI). OTUs present in both abyssal and epipelagic samples were removed, corresponding to 46–58% of OTUs. Further removal of low read count OTUs (n<0.01% of total sequences across all stations) led to a final OTU count of 794, 1219, and 630 for the 18S_V1&2, 18S_V7&8 and mtCOI markers, respectively.

**Figure 2:**
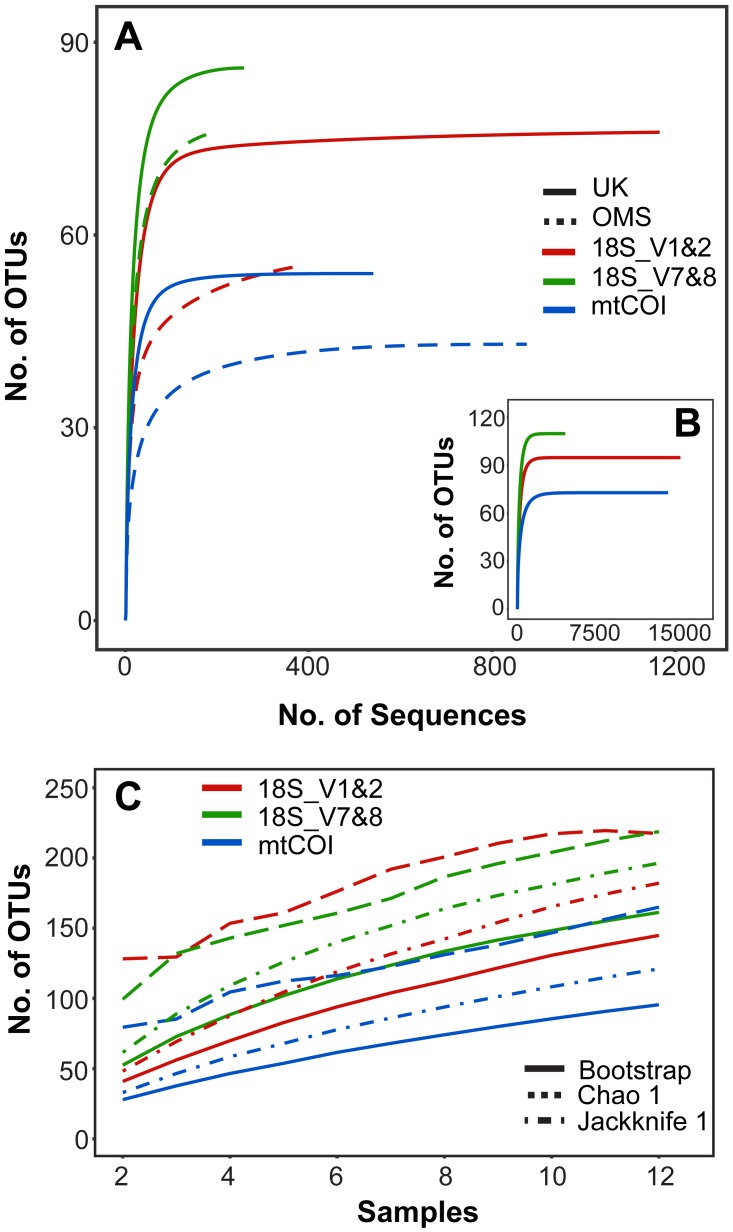
Operational taxonomic unit (OTU) richness for three metabarcoding markers. (A) Sequence-based rarefaction of meroplankton OTU richness compared between markers and sampling strata. (B) Sequence-based rarefaction of meroplankton OTU richness calculated for all 3 markers using pooled UK and OMS sequence data. (C) Chao 1, Jackknife 1 and Bootstrap richness estimators compared between the three markers across all samples (*N* = 12). Figure in C includes both DNA barcoding and metabarcoding data.

Rarefaction curves of meroplankton OTU richness across sequencing coverage by marker for both strata combined suggest sufficient sequencing depth; meroplankton sequence coverage by marker for each individual stratum reached an asymptote in the UK stratum for all three markers and in the OMS stratum for mtCOI (Fig. 2). All three markers consistently detected more OTUs in the UK than the OMS stratum for the meroplankton dataset (Fig. 2, Fig. S1, Table S4). However, none of the species accumulation, Jackknife1, Bootstrap or Chao1 curves across samples reached an asymptote, indicating under-sampling of the local meroplankton assemblage (Fig. 2, Fig. S1). The Good’s coverage index, calculated after subsampling reads to the lowest sequence coverage per site, reached values of 0.96–0.99 across all stations for 18S_V1&2 and mtCOI, whereas 18S_V7&8 didn’t show values over 0.87 (Table S1).

### Metabarcoding–community composition and structure

A total of 95, 110 and 73 meroplankton OTUs were identified with the 18S_V1&2, 18S_V7&8, and mtCOI markers, respectively, accounting for 9–12% of all OTUs detected in the metabarcoding dataset, with the remainder composed of primarily abysso-pelagic copepods. At each sampling site, 18S_V1&2, 18S_V7&8, and mtCOI detected on average 16.8 ± 1.8 (SE), 22.9 ± 2.3 (SE) and 11.6 ± 1.1 (SE) meroplankton OTUs, respectively, and captured a total of 74, 84, and 50 OTUs in the UK and 52, 75, and 40 OTUs in the OMS strata. 93–98% of all meroplankton OTUs were classified to the Order level or lower, while the remaining OTUs were defined as meroplankton based on the taxonomic rank of Class or higher (Tantulocarida, Entoprocta, Actinopterygii, Nemertea). These were listed at their respective taxonomic rank (Table 1) and grouped into ‘Others’ (among several other taxa/OTUs) in most downstream plots and analyses. Within the OTUs classified to the order level or lower, 44–76%, 24–51% and 12–13% were classified to the family, genus and species level, respectively.

**Table 1:** Number of different meroplanktonic taxa identified within each taxonomic rank across 12 phyla, for all three metabarcoding markers. “Combined” refers to the number of different taxa that was observed using all three markers together.

	**18S_V1&2**	**18S_V7&8**	**mt COI**	**Combined**	**18S_V1&2**	**18S_V7&8**	**mt COI**	**Combined**	**18S_V1&2**	**18S_V7&8**	**mt COI**	**Combined**	**18S_V1&2**	**18S_V7&8**	**mt COI**	**Combined**	**18S_V1&2**	**18S_V7&8**	**mt COI**	**Combined**
**Phylum**	**Class**				**Order**				**Family**				**Genus**				**Species**			
Annelida	2	2	2	2	5	6	7	9	10	9	5	12	10	7	4	13	5	6	4	13
Arthropoda	3	3	5	5	4	4	8	8	13	18	13	32	10	12	9	22	1	2	4	6
Bryozoa	1	1	1	1	1	1	1	1	2	2	0	3	2	2	0	3	0	0	0	0
Chordata	2	2	1	2	2	2	6	8	1	1	1	3	1	1	0	2	0	1	0	1
Echinodermata	4	4	3	4	4	4	4	9	4	4	1	6	3	4	1	6	3	4	1	6
Entoprocta	0	0	0	0	0	0	0	0	0	1	0	1	0	0	0	0	0	0	0	0
Mollusca	2	4	2	4	2	4	3	6	2	4	0	5	2	2	0	3	0	0	0	0
Nematoda	1	1	0	1	1	1	0	1	1	1	0	1	0	1	0	1	0	0	0	0
Nemertea	0	1	0	1	0	1	0	1	0	0	0	0	0	0	0	0	0	0	0	0
Platyhelminthes	1	1	0	1	1	1	0	1	0	0	0	0	0	0	0	0	0	0	0	0
Sipuncula	0	1	0	1	0	1	0	1	0	1	0	1	0	0	0	0	0	0	0	0
Xenacoelomorpha	1	0	0	1	1	0	0	1	1	0	0	1	1	0	0	1	0	0	0	0
**Total**	17	20	14	23	21	25	29	46	34	41	20	65	29	29	14	51	9	13	9	26

**Table 2:** Non-parasitic meroplankton OTUs classified to the family, genus, or species level captured by metabarcoding and individual larval barcoding.

**Phylum**	**(Sub-)Class**	**Order**	**Family**	**OTU ID / Species**	**Note**	**Ref.**	**Marker(s)**	%ID
Annelida	Polychaeta	–	Nerillidae	*Nerillidae sp.*		18	18S_V7&8	98.7
Annelida	Polychaeta	–	Nerillidae	*Longipalpa saltatrix*	c	19	18S_V7&8	99.1
Annelida	Polychaeta	–	Travisiidae	*Travisia kerguelensis*		20	18S_V7&8	99.1
Annelida	Polychaeta	–	Travisiidae	*Travisia sp.*		4, 98	18S_V1&2, *18S_V7*&*8*	100.0^**^
Annelida	Polychaeta	Capitellida	Capitellidae	*Capitellidae sp.*		21	18S_V7&8	97.5
Annelida	Polychaeta	Capitellida	Capitellidae	*Capitella capitata*	a, c	5	mtCOI	*
Annelida	Polychaeta	Capitellida	Capitellidae	*Capitella sp.*	a	5,6	*18S_V1*&*2, 18S_V7*&*8*	100.0^**^
Annelida	Polychaeta	Capitellida	Scalibregmatidae	*Neolipobranchius sp.*		22	18S_V1&2	99.2
Annelida	Polychaeta	Eunicida	Dorvilleidae	*Ophryotrocha maculata*	a	23,24	mtCOI	^*^
Annelida	Polychaeta	Eunicida	Dorvilleidae	*Ophryotrocha vivipara*	a	23,24	mtCOI	^*^
Annelida	Polychaeta	Phyllodocida	Chrysopetalidae	*Dysponetus caecus*	e	81,82	–	–
Annelida	Polychaeta	Phyllodocida	Hesionidae	*Neogyptis julii*	e	84	–	–
Annelida	Polychaeta	Phyllodocida	Hesionidae	*Sirsoe sirikos*	a, e	84,85	–	–
Annelida	Polychaeta	Phyllodocida	Polynoidae	*Austropolaria magnicirrata*	e	25,26	18S_V1&2	^*^
Annelida	Polychaeta	Phyllodocida	Polynoidae	*Austropolaria sp.*		25,26	18S_V1&2	84.0^**^
Annelida	Polychaeta	Phyllodocida	Polynoidae	*Macellicephala gloveri*	e	26	–	–
Annelida	Polychaeta	Sabellida	Serpulidae	*Serpulidae sp.*		27,98	18S_V7&8	97.2
Annelida	Polychaeta	Sabellida	Serpulidae	*Protis sp.*		28,29	18S_V1&2	97.6
Annelida	Polychaeta	Sabellida	Siboglinidae	*Osedax frankpressi*	a	1	*All Three*	100.0
Annelida	Polychaeta	Spionida	Chaetopteridae	*Phyllochaetopterus cf.*	a	7,8, 98	18S_V1&2, *18S_V7*&*8*	99.4
Annelida	Polychaeta	Spionida	Chaetopteridae	*Phyllochaetopterus limicolus*		30	18S_V1&2	100.0
Annelida	Polychaeta	Spionida	Spionidae	*Aonides selvagensis*	e	31	18S_V1&2	99.7
Annelida	Polychaeta	Spionida	Spionidae	*Aurospio dibranchiata*		9	*18S_V1*&*2*, 18S_V7&8	99.2
Annelida	Polychaeta	Spionida	Spionidae	*Glandulospio orestes*	e	31	–	–
Annelida	Polychaeta	Spionida	Spionidae	*Laonice sp.*	a, e	9,83, 98	–	–
Annelida	Polychaeta	Terebellida	Fauveliopsidae	*Fauveliopsis scabra*	e	22,86	–	–
Annelida	Polychaeta	Terebellida	Terebellidae	*Amphitrite figulus*	c	32,33	mtCOI	99.00
Arthropoda	Pycnogonida	Pantopoda	Colossendeidae	*Colossendeis sp.*		34,35	mtCOI	89.0^**^
Arthropoda	Copepoda	Cyclopoida	Cyclopinidae	*Cyclopina agilis*	c	2,36	18S_V7&8	100.0
Arthropoda	Copepoda	Cyclopoida	Schminkepinellidae	*Schminkepinellidae sp.*	a	10,11	*18S_V1*&*2*, 18S_V7&8	98.6
Arthropoda	Copepoda	Cyclopoida	Schminkepinellidae	*Cyclopinella sp.*		10	18S_V7&8	100.0
Arthropoda	Copepoda	Cyclopoida	Smirnovipinidae	*Smirnovipinidae sp.*		37	mtCOI	92.1
Arthropoda	Copepoda	Cyclopoida	Speleoithonidae	*Speleoithonidae sp.*	c	2	18S_V7&8	97.5
Arthropoda	Copepoda	Harpacticoida	Aegisthidae	*Pontostratiotes sp.*	a	2,3	*All Three*	100.0
Arthropoda	Copepoda	Harpacticoida	Ameiridae	*Sarsameira sp.*		2,12	18S_V1&2, *18S_V7*&*8*	99.1
Arthropoda	Copepoda	Harpacticoida	Cletodidae	*Cletodidae sp.*		2	18S_V7&8	98.4
Arthropoda	Copepoda	Harpacticoida	Ectinosomatidae	*Bradya sp.*		2,38	18S_V1&2	97.0^**^
Arthropoda	Copepoda	Harpacticoida	Ectinosomatidae	*Parabradya dilatata*		2,39	18S_V1&2	100.0
Arthropoda	Copepoda	Harpacticoida	Idyanthidae	*Idyanthidae sp.*		40	18S_V7&8	100.0
Arthropoda	Copepoda	Harpacticoida	Parameiropsidae	*Parameiropsis sp.*		13	*18S_V1*&*2*, 18S_V7&8	99.2
Arthropoda	Copepoda	Siphonostomatoida	Dirivultidae	*Dirivultidae sp.*	b	2,41	18S_V1&2	97.5
Arthropoda	Eucarida	Decapoda	Xanthidae	*Xantho sp.*	c	6,35	mtCOI	99.0
Arthropoda	Hexanauplia	Pedunculata	Scalpellidae	*Scalpellidae sp.*		43	mtCOI	95.0
Arthropoda	Malacostraca	Amphipoda	Alicellidae	*Paralicella caperesca*	d	44	mtCOI	100.0
Arthropoda	Peracarida	Amphipoda	Caprellidae	*Caprellidae sp.*		45,46	mtCOI	94.6
Arthropoda	Peracarida	Amphipoda	Caprellidae	*Caprella sp.*		45,47	mtCOI	100.0
Arthropoda	Peracarida	Amphipoda	Uristidae	*Uristidae sp.*	d	48	mtCOI	94.1
Arthropoda	Peracarida	Amphipoda	Uristidae	*Abyssorchomene chevreuxi*	d	49,50	mtCOI	99.7
Arthropoda	Peracarida	Amphipoda	Valettiopsidae	*Valettiopsidae sp.*	d	51	mtCOI	94.1
Arthropoda	Peracarida	Isopoda	Ligiidae	*Ligiidae sp.*	c	52	mtCOI	97.0
Arthropoda	Peracarida	Isopoda	Macrostylidae	*Macrostylis sp.*		9,53	18S_V7&8	99.7
Arthropoda	Peracarida	Isopoda	Munnopsidae	*Disconectes sp.*		54	18S_V1&2	99.5
Arthropoda	Peracarida	Isopoda	Munnopsidae	*Munnopsurus sp.*		54,55	18S_V1&2	99.7
Arthropoda	Peracarida	Isopoda	Munnopsidae	*Tytthocope sp.*		54,55	18S_V1&2	99.7
Bryozoa	–	Ctenostomatida	Paludicellidae	*Paludicella sp.*	c	58	18S_V7&8	^*^
Bryozoa	–	Ctenostomatida	Triticellidae	*Triticella sp.*		59	18S_V7&8	99.0^**^
Echinodermata	Asteroidea	Paxillosida	Porcellanasteridae	*Porcellanaster ceruleus*		14	*18S_V7*&*8*, mtCOI	100.0
Echinodermata	Asteroidea	Valvatida	Asterinidae	*Patiriella regularis*	c	60	18S_V1&2	99.4
Echinodermata	Ophiuroidea	Ophiurida	Ophiuridae	*Ophionotus victoriae*		61	18S_V7&8	100.0
Echinodermata	Ophiuroidea	Ophiurida	Ophiuridae	*Ophiopleura borealis*		62	18S_V1&2	100.0
Echinodermata	Echinoidea	Arbacioida	Arbaciidae	*Arbaciidae sp.*		63	18S_V1&2	98.1
Echinodermata	Echinoidea	Temnopleuroida	Toxopneustidae	*Lytechinus variegatus*	c	64	18S_V7&8	100.0
Echinodermata	Holothuroidea	Elasipodida	Deimatidae	*Oneirophanta setigera*		14	*18S_V1*&*2*, 18S_V7&8	100.0
Mollusca	Bivalvia	Myoida	Xylophagidae	*Xylophagidae sp.*	a, e	24,80	–	–
Mollusca	Bivalvia	Nuculanoida	Yoldiidae	*Yoldiella sp.*	e	79	–	–
Mollusca	Bivalvia	Pectinoida	Propeamussiidae	*Propeamussium sp.*	e	77,78	–	–
Mollusca	Gastropoda	Vetigastropoda	Calliostomatidae	*Calliostomatidae sp.*	e	66	18S_V7&8	97.0
Mollusca	Gastropoda	Vetigastropoda	Calliostomatidae	*Calliostoma sp.*	e	75,76	–	–
Mollusca	Gastropoda	Vetigastropoda	Calliotropidae	*Calliotropidae sp.*	e	6	18S_V7&8	98.8
Mollusca	Gastropoda	Vetigastropoda	Larocheidae	*Bathyxylophila sp.*	a, e	72,73	–	–
Mollusca	Gastropoda	Vetigastropoda	Seguenziidae	*Fluxinella sp.*	e	67	18S_V1&2	86.0^**^
Mollusca	Gastropoda	Vetigastropoda	Seguenziidae	*Ventsia tricarinata*	a, e	73,74	–	–
Mollusca	Polyplacophora	Chitonida	Ischnochitonidae	*Ischnochiton sp.*		15	*18S_V1*&*2,* 18S_V7&8	97.2
Mollusca	Scaphopoda	Gadilida	Gadilinidae	*Gadila sp.*		6	18S_V7&8	100.0
Nematoda	Enoplea	Enoplida	Phanodermatidae	*Phanodermatidae sp.*		68	18S_V1&2	99.7
Nematoda	Enoplea	Enoplida	Phanodermatidae	*Phanodermopsis sp.*		68	18S_V7&8	99.4
Sipuncula	Phascolosomatidea	Phascolosomatida	Phascolosomatidae	*Phascolosomatidae sp.*	e	69,70	18S_V7&8	97.8
Sipuncula	Phascolosomatidea	Phascolosomatida	Phascolosomatidae	*Phascolosoma sp.*	e	69,70	–	–
Xenacoelomorpha	–	Acoela	Mecynostomidae	*Childia sp.*	c	71	18S_V1&2	100.0

**Notes.**

a Bone-Burrowing/Chemosynthetic Habitat.

b Hard Substrate Habitat.

c Dubious Report (shallow water or anchihaline cave), due to an absence of close relatives in reference databases.

d Scavenging.

e Detected by larval barcoding.

Marker(s) = Identity of marker(s) capturing the OTU. If captured by multiple markers, the marker with the highest blastn percent ID is italicized.

%ID = highest blastn percent ID of all markers.

‘-’ = OTU was only detected by larval barcoding.

* Best taxonomic inference made using SAP (no %ID).

** Best taxonomic inference with Wang & SILVA (number indicates posterior probability and not %ID).

Ref. = Reference(s). References are listed in File S2.

**Figure 3:**
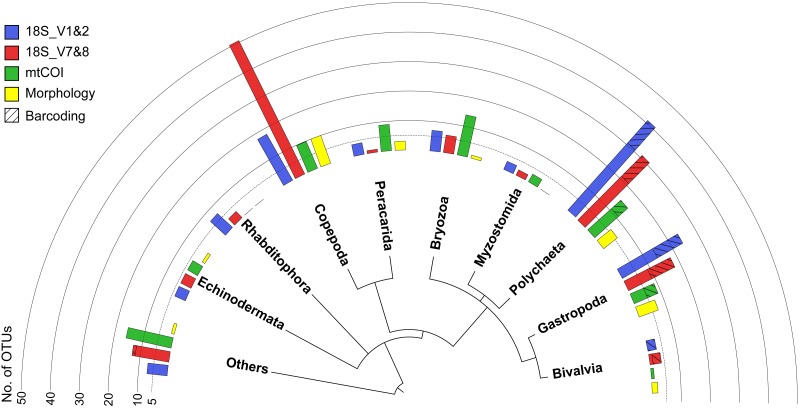
Overview phylogram of meroplankton diversity detected across all methods. Results shown for metabarcoding using three markers (blue, red, green), DNA barcoding of individual larvae (cross hatching), and microscopy (Morphology; yellow). Bar graphs indicate the number of OTUs detected for each of the 10 taxonomic groups. Others (12.9% total) includes Nematoda (0.7% of all meroplankton OTUs combining the three metabarcoding datasets), Tantulocarida (0.7%), Tunicata (2.2%), Vertebrata (3.6%), Chitonida (0.7%), Xenacoelomorpha (0.4%), Decapoda (0.4%), Pedunculata (1.1%), Pycnogonida (0.4%), Entoprocta (1.4%), Nemertea (0.7%), Scaphopoda (0.4%), and Sipuncula (0.4%).

All three markers detected a similar overall composition of the meroplankton assemblage, with representatives identified from 12 phyla, comprised of the family- and genus-richest groups Annelida, Arthropoda, and Echinodermata, as well as the phyla Bryozoa, Chordata, Entoprocta, Mollusca, Nematoda, Nemertea, Platyhelminthes, Sipuncula, and Xenacoelomorpha. Within those phyla, the metabarcoding approach detected a total of 23 classes, 46 orders, 65 families, 51 genera, and 26 species (Tables 1 and 2). However, the three metabarcoding markers performed differently in detecting richness and diversity of the meroplanktonic assemblage (Fig. 3). 18S_V1&2 detected the highest number of polychaete (31), gastropod (14) and platyhelminthes (seven) OTUs, while 18S_V7&8 recovered the highest diversity of copepod (five) OTUs. mtCOI identified the highest number of ‘Other’ (16) and bryozoan (14), as well as peracarid (nine) OTUs (Fig. S2). The taxonomic group ‘Other’ included, combined across all markers, tantulocarids (two OTU), tunicates (six OTUs), pycnogonids (one OTU), decapods (one OTU), pedunculates (three OTUs), vertebrates (10 OTUs), xenacoelomorphs (one OTU), entoprocts (four OTUs), chitons (two OTUs), nematodes (one OTU), nemerteans (two OTUs), scaphopods (one OTU) and sipunculans (one OTU) (Table 2). At a higher taxonomic resolution, within the 65 detected families, 41 were unique to one of the three markers, 18 were detected by two markers and six were shared between all three (Aegisthidae, Clausidiidae, Schminkepinellidae—copepods; Capitellidae, Siboglinidae—polychaetes; Endomyzostomidae; Fig. S3A). Of the 51 captured genera, 34 were unique to one of the markers, 13 were identified by two markers, and four (*Pontostratiotes, Hemicyclops*—copepods; *Capitella, Osedax*—polychaetes) were detected by all three (Fig. S3B). OTU classification to the species level was most successful with 18S_V7&8 (13 species, 13.6% of OTUs classified to species level) followed by mtCOI (9 species, 12.3%) and 18S_V1&2 (9 species, 11.6%) (Table 1). All three markers therefore uncovered great taxon richness and diversity, including groups that remained undetected by the other two markers, as well as several taxa that had previously not been reported in the deep Pacific nor any other deep-sea ecosystem (Tables 2 and 3).

**Table 3:** Commensal/Parasitic meroplankton OTUs at the family, genus, or species level captured by metabarcoding and individual barcoding, including information on the host associations.

**Phylum**	**(Sub-)Class**	**Order**	**Family**	**OTU ID / Species**	**Host Association**	**Ref.**	**Marker(s)**	**%ID**
Annelida	–	Myzostomida	Endomyzostomatidae	*Endomyzostomatidae sp.*	Endoparasites of crinoids.	16	mtCOI	98.0
Annelida	–	Myzostomida	Endomyzostomatidae	*Endomyzostoma cysticolum*	Endoparasite of crinoids.	17	18S_V7&8	99.1
Annelida	–	Myzostomida	Endomyzostomatidae	*Endomyzostoma sp.*	Endoparasites of crinoids.	16	18S_V1&2	100.0^**^
Arthropoda	Copepoda	Cyclopoida	Anchimolgidae	*Anchimolgidae sp.*	Associates of scleractinian corals.	2	18S_V1&2	97.5
Arthropoda	Copepoda	Cyclopoida	Clausidiidae	*Hemicyclops sp.*	Associates of various marine invertebrates, incl. sponges, molluscs, cnidarians, and others.	2	*18S_V1*&*2*, 18S_V7&8, mtCOI	97.3
Arthropoda	Copepoda	Cyclopoida	Lichomolgidae	*Lichomolgidae sp.*	Associates or parasites of marine invertebrates, incl. molluscs, echinoderms and ascidians.	2	18S_V7&8	96.2^*^
Arthropoda	Copepoda	Cyclopoida	Myicolidae	*Myicolidae sp.*	Parasites of bivalve molluscs and sometimes pests of commercially important species.	2	18S_V7&8	98.4
Arthropoda	Copepoda	Cyclopoida	Rhynchomolgidae	*Critomolgus sp.*	Associates of Ophiuroidea, Crinoidea, Nudibranchia, Alcyonacea, Actinaria, and Pennatulacea.	2	18S_V7&8	98.7
Arthropoda	Copepoda	Cyclopoida	Synapticolidae	*Synapticolidae sp.*	Associates of echinoderms.	2	18S_V7&8	98.7
Arthropoda	Copepoda	Cyclopoida	Synapticolidae	*Scambicornus sp.*	Associates of holothurians.	2	*18S_V1*&*2*, 18S_V7&8	97.5
Arthropoda	Copepoda	Siphonostomatoida	Asterocheridae	*Asterocheridae sp.*	Associates or parasites of marine invertebrates, incl. sponges, cnidarians, and echinoderms.	2	18S_V1&2	98.3
Arthropoda	Copepoda	Siphonostomatoida	Eudactylinidae	*Eudactylinidae sp.*	Parasites of elasmobranch fishes or other fish groups.	2	*18S_V1*&*2*, 18S_V7&8	97.2
Arthropoda	Copepoda	Siphonostomatoida	Eudactylinidae	*Nemesis sp.*	Associates of sharks.	42	18S_V7&8	99.1
Arthropoda	Copepoda	Siphonostomatoida	Nicothoidae	*Rhizorhina soyoae*	Associates or parasites of amphipods, isopods, tanaids, and members of Leptostraca.	2	*18S_V7*&*8, mtCOI*	100.0
Arthropoda	Tantulocarida	–	Deoterthridae	*Arcticotantulus sp.*	Ectoparasites of harpacticoids (only reported from the White and Greenland Sea).	56,57	18S_V7&8	92.0^**^
Entoprocta	–	–	Loxosomatidae	*Loxosomatidae sp.*	Epibionts of polychaetes, bryozoans, poriferans, and other invertebrates.	65	18S_V7&8	98.0

**Notes.**

Marker(s) = Identity of marker(s) capturing the OTU. If captured by multiple markers, the marker with the highest blastn percent ID is italicized.

%ID = highest blastn percent ID of all markers.

* best taxonomic inference made using SAP.

** best taxonomic inference with Wang & SILVA (number indicates posterior probability and not %ID).

Ref. = Reference(s). References are listed in File S2.

**Figure 4:**
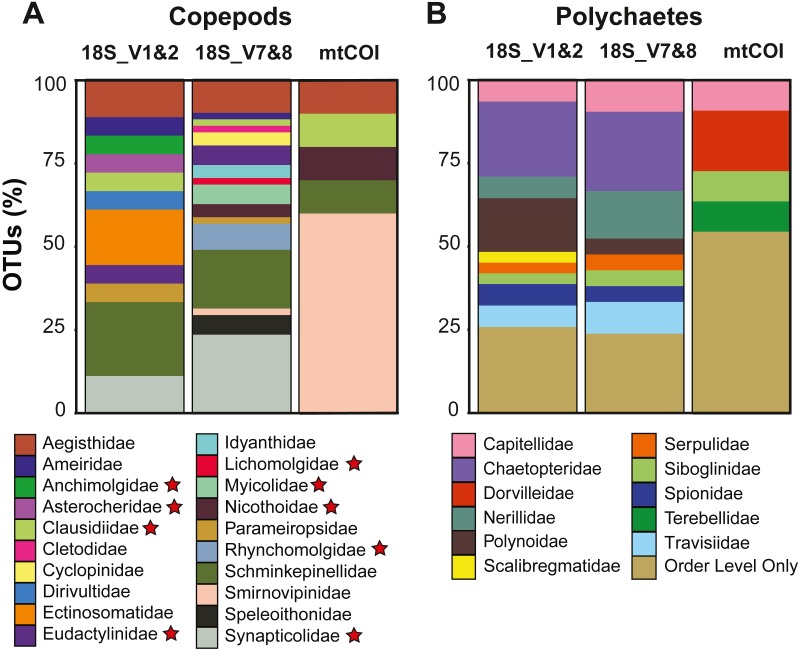
Diversity captured within meroplanktonic Copepoda and Polychaeta study-wide. Relative operational taxonomic unit (OTU) proportion of (A) copepod and (B) polychaete families compared between three metabarcoding markers. Parasitic/commensal copepod families are marked with a star.

Copepods dominated the meroplankton diversity captured with the 18S_V7&8 marker, accounting for 46.4% of all OTUs (Fig. S2). Across all markers, 21 copepod families were sampled, including both meiobenthic and parasitic/commensal taxa (Tables 2 and 3, Fig. 4). The families Schminkepinellidae, Synapticolidae and Smirnovipinidae comprised the highest proportion of OTUs across all markers (Fig. 4). Within epibenthic/meiobenthic groups, families accounting for more than 10% of all copepod OTUs included Schminkepinellidae (22.2%), Ectinosomatidae (16.7%) and Aegisthidae (11.1%) for 18S_V1&2, Schminkepinellidae (17.6%) for 18S_V7&8, and Smirnovipinidae (60%) for mtCOI (Fig. 4). Although none of the families were reported across all sampling sites, Aegisthidae, Schminkepinellidae, Ectinosomatidae, Cyclopinidae, Speleoithonidae and Smirnovipinidae were reported from at least six of the twelve sampling sites. Within the parasitic copepods, the Synapticolidae (11.1–23.5%, 18S), echinoderm associates (Table 3), reached more than 10% of the copepod OTUs and they were the only family to be reported across all sampling sites (18S_V7&8). However, Anchimolgidae, Synapticolidae, Myicolidae, Rhynchomolgidae and Clausidiidae OTUs were recovered from at least 6 of the 12 sampling sites.

Polychaetes dominated the meroplankton assemblage captured by the 18S_V1&2 marker, accounting for 32.6% of all OTUs (Fig. S2). Across all markers, this study sampled eleven families of polychaetes (Table 2, Fig. 4), with the families Chaetopteridae (22.6–23.8%, 18S), Polynoidae, (16.1%, 18S_V1&2), Nerillidae (14.3%, 18S_V7&8) and Dorvilleidae (18.2%, mtCOI) accounting for more than 10% of all polychaete OTUs (Fig. 4). All of the polychaete families were detected at less than half of the twelve sampling sites (<6 sites). For all three markers, the largest fraction (23.8–54.5%) of polychaete OTUs could be classified only to Order level due to an absence of close relatives in reference databases, including the benthic orders Spionida, Capitellida, Eunicida, Ophellida and Sabellida (Fig. 4).

In terms of read abundance, copepods and polychaetes together accounted for the majority (56.5%) of sequence reads, dominating the community captured by 18S_V7&8 (Fig. S2). Using 18S_V1&2, read abundance in the meroplankton assemblage was more evenly distributed, with copepods and polychaetes accounting for a combined 26.3% of all sequence reads, followed by rhabditophorans (18.5%), peracarids (17.7%) and gastropods (15.8%) (Fig. S2). Within the meroplankton assemblage detected by mtCOI, polychaetes dominated read abundances representing 32.5% of all sequences and comprising more than twice as many reads as peracarids (15.5%), copepods (12.9%), ‘Others’ (12.1%) and echinoderms (11.9%) (Fig. S2).

### DNA barcoding of Individual Larvae

Including all 42 larval specimens, 90.5% were classified to family, 61.9% to the genus, and 31.0% to the species level, resulting in the identification of 13, 15, and 8 different families, genera, and species, respectively, and a total of 21 distinct taxa (Table S5). Polychaetes (*N* = 23) were the most family-, genus-, and species-rich taxon, including the families Spionidae (*N* = 10), Chrysopetalidae (*N* = 4), Hesionidae (*N* = 3), Polynoidae (*N* = 3) and Fauveliopsidae (*N* = 1) (Table S5). Within those families, five of the eight detected genera (*Austropolaria, Dysponetus, Fauveliopsis, Glandulospio, Laonice, Macellicephala, Neogyptis* and *Sirsoe*) and four of seven species were represented by a single specimen (Table S5). Two sipunculans were sampled and assigned to the family Phascolosomatidae, genus *Phascolosoma*. Each of the three bivalve specimens were assigned to a different family (Propeamussidae, Yoldiidae and Xylophagidae) and two of them to a genus (*Propeamussium, Yoldiella*), but none to the species level (Table S5). Among the 14 gastropods, 78.6% were assigned to one of four families, comprising the Larocheidae (*N* = 2), Calliotropidae (*N* = 3), Seguenziidae (*N* = 5) and Calliostomatidae (*N* = 1). Within those families, 72.7% of the specimens were assigned to a genus, which included the genera *Fluxinella* (*N* = 4), *Bathyxylophila* (*N* = 2), *Ventsia* (*N* = 1) and *Calliostoma* (*N* = 1), and a single specimen was classified to the rank of species (*Ventsia tricarinata*) (Table S5).

Comparing the barcoding of individual larvae and metabarcoding results, the barcoding dataset contained seven families, twelve genera, and six species that were not detected by metabarcoding (Tables 2, S5). These included the gastropod family Larocheidae, the bivalve families Propeamussiidae, Yoldiidae and Xylophagidae, as well as the polychaete families Chrysopetalidae, Hesionidae and Fauveliopsidae (Table 2), and apart from the polychaetes, each taxon was represented by a single sorted specimen across all sites. Also, sequences from only seven (17.1%, 18S_V1&2), three (7.7%, 18S_V7&8) and one (6.25%, mtCOI) specimen fell within 99% (18S) and 97% (mtCOI) similarity of an OTU representative sequence from the metabarcoding data, further emphasizing the distinctiveness of the two datasets (Table S5). Of the remaining specimens, 14, 16 and four polychaetes were within 99% (18S) and 97% (mtCOI) sequence similarity to another barcoded specimen, for the 18S_V1&2, 18S_V7&8 and mtCOI markers, respectively, and were subsequently treated as the same OTU for that marker (Table S5). Hence, sorting larger larval specimens prior to metabarcoding essentially removed 23 (10 polychaetes, three bivalves and 10 gastropods), 23 (10 polychates, three bivalves, nine gastropods and 1 sipunculid) and eight (four polychaetes and four gastropods) OTUs from the 18S_V1&2, 18S_V7&8 and mtCOI metabarcoding datasets, respectively (Fig. 3, Table S5). Conversely, the gastropod OTUs *Calliotropidae sp.* (*N* = 1, 18S_V7&8) and *Vetigastropoda sp.* (*N* = 3, 18S_V1&2; *N* = 2, 18S_V7&8) and the polychaete OTUs *Polynoidae sp*. (*N* = 2, 18S_V1&2) and *Capitella sp.* (*N* = 2, 18S_V1&2; *N* = 1, mtCOI) were shared between both datasets.

### Spatial variability in the larval assemblage

**Figure 5:**
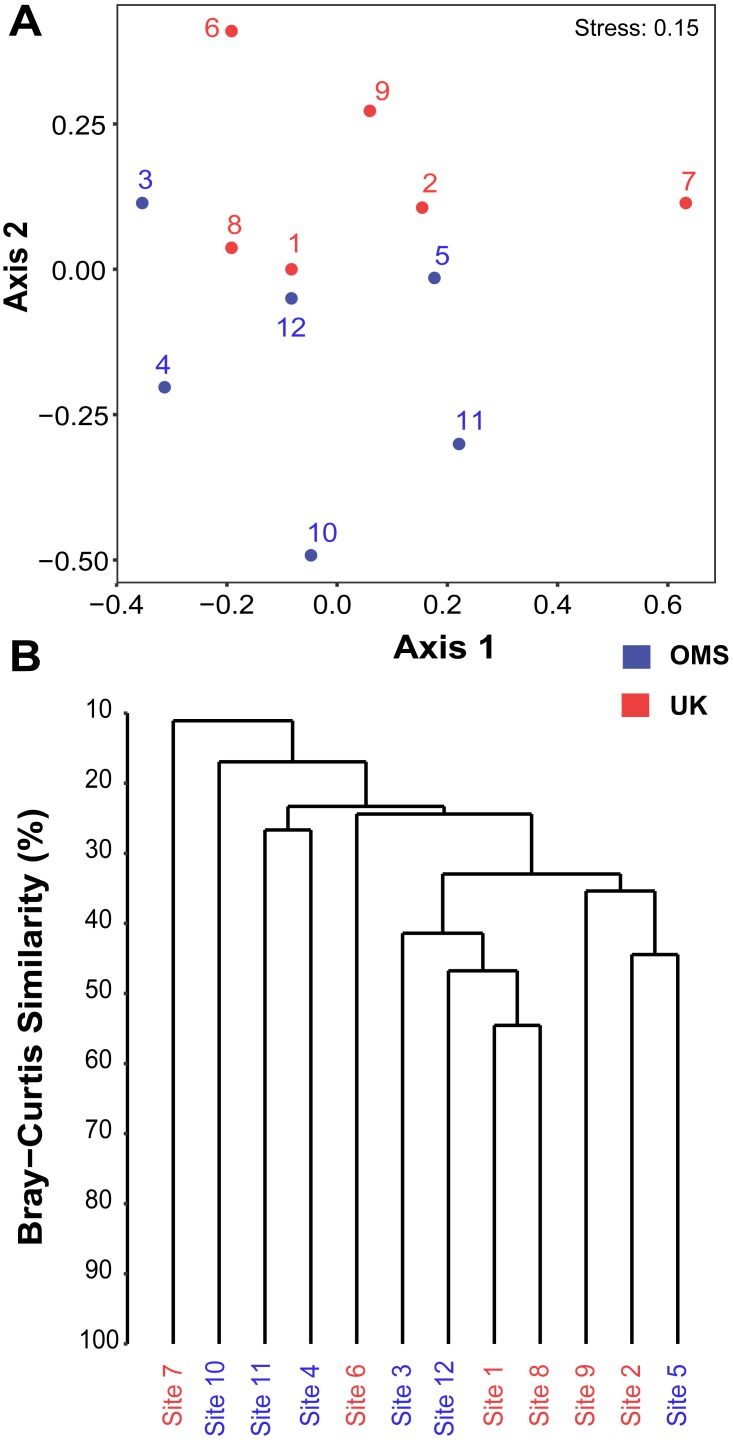
Spatial patchiness and structure of the meroplankton assemblage captured by metabarcoding across 12 abyssal sites. The non-parametric multidimensional scaling (NMDS) plot (A) and cluster dendrogram (B) were constructed for the 18S_V1&2 marker. The NMDS plot shows the placement of 12 abyssal sites (UK1 +OMS1) in ordination space, colored by strata. The dendrogram is based on a hierarchical cluster analysis using group average linkage and the Bray–Curtis distance measure. OTU sequence abundance was transformed to presence/absence prior to both analyses.

**Figure 6:**
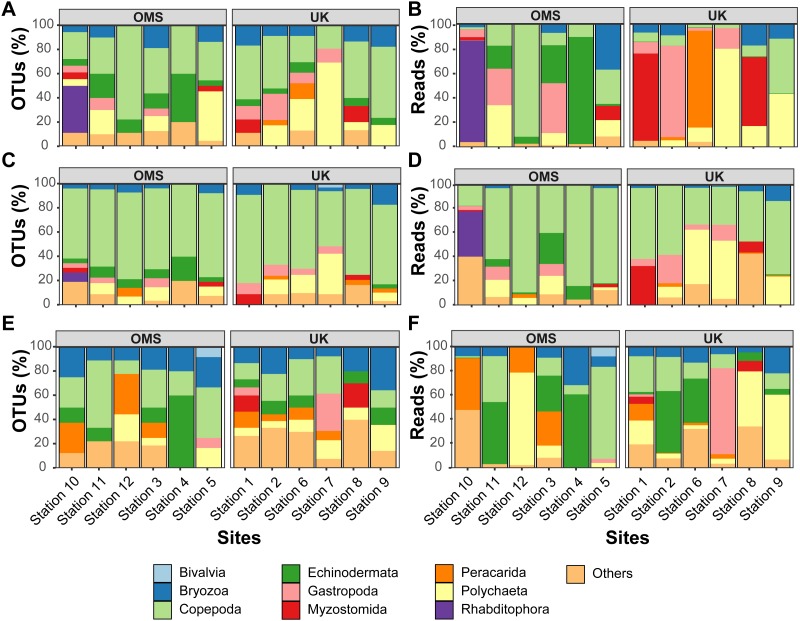
Variability of relative operational taxonomic unit (OTU) and sequence read abundance (%) captured by metabarcoding across 12 abyssal sites. Relative OTU (A, C, E) and sequence read (B, D, F) abundance detected by three metabarcoding markers is shown for 10 dominant meroplankton groups at each sampling site within the OMS and UK strata. Amplicons originate from markers (A–B) 18S_V1&2, (C–D) 18S_V7&8, and (E–F) mtCOI. Nematoda (0.7% of all meroplankton OTUs combining the three metabarcoding datasets), Tantulocarida (0.7%), Tunicata (2.2%), Vertebrata (3.6%), Chitonida (0.7%), Xenacoelomorpha (0.4%), Decapoda (0.4%), Pedunculata (1.1%), Pycnogonida (0.4%), Entoprocta (1.4%), Nemertea (0.7%), Scaphopoda (0.4%), and Sipuncula (0.4%) were grouped into Others (12.9% total).

The predominant pattern in the composition and structure of the meroplankton assemblage was high site-to-site variability within and across the UK and OMS strata. For all three markers, 42.7–63.0% of the detected meroplankton OTUs were unique to a single site, while 5.5–7.4% of the OTUs occurred at six or more sites including the copepod families Anchimolgidae, Schminkepinellidae, Aegisthidae, Myicolidae, Speleoithonidae, Clausidiidae, Smirnovipinidae, and echinoderms Arbaciidae and Toxopneustidae. Also, <2.0% of meroplankton OTUs were observed at all UK or OMS sites, while 94.7–97.3% and 95.5–97.9% occurred at three or fewer UK or OMS sites, respectively. Further illustrating the relatively low taxonomic overlap in the meroplankton assemblages at each site, Bray–Curtis similarity between sites never exceeded 54.7% for any marker (Figs. 5, S4). Similarly, considerable spatial heterogeneity was observed for relative OTU and sequence abundance in both 18S_V1&2 and mtCOI results (Figs. 6, S5), while little variability across sites was found with 18S_V7&8 due in large part to the substantial dominance of copepods.

In addition to considerable between-site differences across all sites, metabarcoding also captured variation in meroplankton composition and structure between the UK and OMS strata. Across the three markers, 55.5–72.6% of the meroplankton OTUs were detected in only one of the two strata, while 60.0–63.3% of those occurring in both strata were captured at only one site in each area. The differences in composition and structure were further supported by relative OTU and sequence abundance results using the 18S_V1&2 and mtCOI markers. Mean OTU proportions captured by 18S_V1&2 and mtCOI were markedly dissimilar between the UK vs. the OMS stratum for the four OTU-richest taxa detected by each marker (i.e., copepods (both), polychaetes (18S_V1&2) rhabditophorans (18S_V1&2) and bryozoans; Figs. 6, S5). The mean relative sequence abundance of rhabditophorans (0.0% vs. 13.7%), peracarids (13.5% vs. 0.0%), and myzostomids (21.1% vs. 2.3%) for 18S_V1&2, as well as gastropods (12.1% vs. 0.6%) for mtCOI, also were noticeably different between the UK and OMS stratum, respectively (Figs. 6, S5).

Despite the observed differences in the meroplankton assemblage between the UK and OMS strata and marginal separation in the NMDS plot (Figs. 5, S4), statistical analyses of community structure and composition failed to detect significant spatial patterns separating the two strata (OMS, UK), likely as a result of both the high between-site variability within each claim area and incomplete sampling of the larval assemblage. ANOSIM, PERMANOVA using distance matrices, and MRPP analyses did not support distinct grouping into the two sampled strata based on similarity in community composition after data transformation into presence/absence of OTUs and indicated large within-strata/between-site variation (Table S4). Correspondingly, differences in OTU diversity and evenness indices between the OMS and UK stratum were non-significant (Table S4), despite persistent trends towards higher meroplankton richness in the UK stratum at each site for all three markers (Figs. 2, S1, Table S1). The lack of spatial patterning of meroplanktonic assemblages into clear UK and OMS strata was also observed in results of the hierarchical cluster analyses (Figs. 5, S4). Similarly, results using non-transformed OTU sequence abundances also failed to support a spatial clustering of the sites into UK vs. OMS strata (Table S4). Finally, spatial (Euclidean) distance between stations was not a significant predictor of community dissimilarity, as revealed by Mantel tests (Table S4).

### Comparison of molecular and morphological analyses

**Figure 7:**
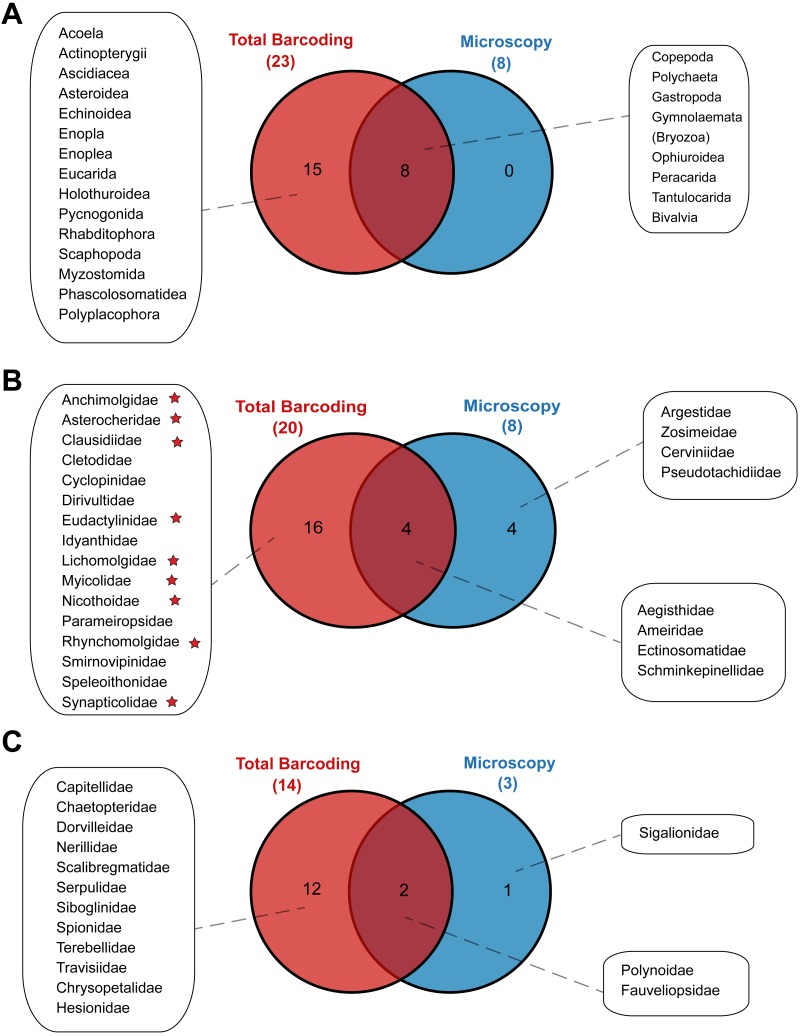
Venn diagrams depicting taxonomic overlap in meroplankton diversity recovered by microscopy and total barcoding. Numbers in parentheses represent the total number of (A) meroplankton classes, (B) meroplanktonic copepod families and (C) meroplanktonic polychaete families captured by each method. Copepod families marked with a star are parasitic/commensal.

Metabarcoding and DNA barcoding of individual larvae combined (termed ‘Total barcoding’) detected far higher diversity within the meroplankton assemblage (Table 2, Fig. 3) than was found by morphological analysis alone (Kersten, Smith & Vetter, 2017). Total barcoding captured seven phyla and twelve classes that were missed by microscopy, as well as a 2.7–4.3 times higher meroplankton OTU richness. In sum, 23 meroplanktonic classes were identified by Total barcoding, including the eight groups that were also detected by microscopy (Figs 3, 7), which consisted of Polychaeta, Bivalvia, Gastropoda, Peracarida, Gymnolaemata (Bryozoa), Tantulocarida, and Ophiuroidea, arguably all classic meroplankton taxa, as well as Copepoda. The remaining 15 classes, which included several rarely reported meroplankton groups such as Myzostomida, Polyplacophora or Scaphopoda, were not seen under the microscope (Figs. 3, 7).

Within the copepods, both microscopy and metabarcoding detected the families Ameiridae, Schminkepinellidae, Ectinosomatidae, and Aegisthidae (Tables 2, S6, Fig. 7). Metabarcoding found an additional 16 copepod families, the majority of which were parasitic/commensal, that were undetected by microscopy, including Clausidiidae, Synapticolidae, Nicothoidae and Parameiropsidae (Table 3). Microscopy discovered four families that were absent or weren’t detected at comparable taxonomic resolution in the metabarcoding dataset (Argestidae, Zosimeidae, Cerviniidae and Pseudotachidiidae). These latter families could be represented in the 3.9–4.4% of copepod OTUs that were not classified beyond Copepoda or Harpacticoida across the three markers, due to an absence of reference sequences (Fig. 7). At the genus level, one of the two copepod genera detected by microscopy, *Barathricola* (family Schminkepinellidae), was absent in metabarcoding classifications despite detecting the family (presumably no reference sequence), while the other genus, *Pontostratiotes* (family Aegisthidae), was detected by all three metabarcoding markers (Table 2, Table S6).

Within polychaetes, microscopy, metabarcoding and DNA barcoding all detected the family Polynoidae and both barcoding and microscopy discovered the family Fauveliopsidae, while Total barcoding uncovered an additional 12 families, including Siboglinidae, Travisiidae, Capitellidae, Spionidae, and Dorvilleidae, and microscopy identified one other family (Sigalionidae) undetected by the other comparative analyses (Fig. 7).

## Discussion

Although abyssal plains are among the least explored marine ecosystems on our planet, significant regions of the deep seafloor in the Pacific are potentially at risk due to polymetallic nodule mining. Larval dispersal must play a key role in the resilience of abysso-benthic populations, for example by facilitating recolonization following mining disturbance, yet this is the first study to investigate larval diversity over the abyssal plains in the Pacific (with Kersten, Smith & Vetter, 2017), and the first to use metabarcoding methods to study larvae in any deep ocean habitat. Our results suggest that larval assemblages in the benthic boundary layer can be highly diverse at the phylum level (Tables 1, 2), and are spatially patchy at the oceanographic mesoscale (10s of km). An assemblage of parasitic and commensal copepods and worms, that were in some cases spatially quite widespread in our samples, have not been previously reported in the deep ocean (Table 3). We also sampled larvae of several residents of deep chemosynthetic/organic-rich habitats, including whale-fall, wood-fall and hydrothermal vent taxa, such as *Osedax*, *Xylophaga* and *Ventsia*, respectively (Table 2).

The meroplankton richness captured by metabarcoding, with representatives from 12 phyla, 23 classes, 46 orders, and 65 families, included many organisms that have been previously reported from the CCZ or other deep-sea regions in the Pacific. Taxa such as *Porcellanaster ceruleus*, *Oneirophanta setigera*, *Ophryotrocha,* or *Yoldiella* have previously been observed as adults in related studies of megafauna/macrofauna from the same CCZ claim areas, while others (*Parameiropsis*, *Laonice, Macrostylis*) were recorded in other areas of the eastern and central CCZ (Cho, Wi & Suh, 2016; Amon et al., 2017b; Amon et al., 2017c; De Smet et al., 2017; Wiklund et al., 2017; Table 2). Sampled taxa that are known to occur in other areas of the deep Pacific include numerous polychaetes and several gastropods, as well as groups that are more infrequently reported as part of the meroplankton, such as scaphopods, entoprocts (parasitic/commensal), or myzostomids (parasitic) (Table 1, Fig. 3). Finally, we also provide the first reports of an abyssal distribution in the Pacific for several shallow water taxa, as well as taxa previously only recorded from other ocean basins, including for example the polychaete genus *Austropolaria* or the echinoderm family Arbaciidae, which are likely represented by undescribed members of those taxa (Table 2). Nevertheless, species accumulation curves and species richness estimators (Jackknife1, Chao1, Bootstrap) indicated under-sampling of the larval diversity at each site, suggesting that additional plankton pump deployments or increased filtration volumes would be required to fully capture the meroplankton diversity (Figs. 2, S1).

Polychaetes and copepods are dominant components of the macrofauna and meiofauna in the abyssal benthos (e.g., Ahnert & Schriever, 2001; De Smet et al., 2017; Wilson, 2017), and were the richest taxonomic groups detected across all three metabarcoding markers in this study. The polychaete larval assemblage included several typical abyssal benthic polychaetes, such as the families Capitellidae, Acrocirridae, Travisiidae, Spionidae, Scalibregmatidae, Polynoidae, Terebellidae or Serpulidae, all of which have been previously sampled in the CCZ (Table 2, Fig. 4). Most of these polychaete families are known epifauna and/or infauna of the soft sediment community; however, members of the family Serpulidae are commonly found on nodules (Amon et al., 2017c) and other hard substrates (Amon et al., 2016; Vanreusel et al., 2016). Most of the polychaete families detected also are deposit-feeders (Jumars, Dorgan & Lindsay, 2015), with the exception of polynoids, siboglinids (*Osedax*) and dorvilleids, which may be carnivores, omnivores, and/or bone feeders (Jumars, Dorgan & Lindsay, 2015). Polychaetes serve important roles in recycling and bioturbation of sediments, as well as in the burial of organic matter (Hutchings, 1998); these ecosystem functions make them an important faunal component to document in baseline surveys of the CCZ. Apart from the typical abyssal families, we also sampled polychaetes that are known from chemosynthetic/organic-rich habitats, e.g., hydrothermal vents and whale falls, including the species *Osedax frankpressi* and *Capitella capitata*, and the genera *Ophryotrocha*, *Phyllochaetopterus*, *Protis* and *Laonice* (Table 2). The presence of meroplankton of these taxa over the abyssal plain may indicate the presence of whale falls or other reducing/organic-rich habitats within dispersal distance of the sampling sites (whale falls have been observed within the CCZ; Smith et al., 2015; Amon et al., 2017a), consistent with these taxa dispersing across the abyssal plain via habitat ‘stepping stones’ (Vrijenhoek, 2010; Smith et al., 2015).

Our metabarcoding approach also captured twenty families of copepods, including both parasitic/commensal and meiofaunal groups (Fig. 4, Table 3). Copepods are common parasitic or commensal associates of many host taxa with deep sea representatives (Boxshall & Halsey, 2004; Boxshall, 1998). In particular, the families Anchimolgidae, Asterocheridae, Clausidiidae, Eudactylinidae, Lichomolgidae, Myicolidae, Nicothoidae, Rynchomolgidae, and Synapticolidae sampled here have been observed on a variety of hosts, including corals, sponges, cnidarians, echinoderms, polychaetes, molluscs, crustaceans, and fishes. Yet apart from Clausidiidae, Rhynchomolgidae and Lichomolgidae, members of these families display host specificity and are associated with only one of these groups (Table 3). Nevertheless, very little is known about the distribution, host specificity or life history of parasitic copepods in the deep-sea (De Buron & Morand, 2004; Boxshall & Halsey, 2004). Presumably, we lack information about parasitic copepods at great depths due to their inconspicuousness, as well as the fact that the deep-sea remains highly under-sampled (De Buron & Morand, 2004). The parasitic copepods reported here were almost certainly collected as pelagic nauplii, the life history phase that disperses and locates new hosts. Approximately 50% of the specimens collected by our plankton pumps were nauplii (Kersten, 2015); however, nauplii are extremely challenging to identify morphologically (Jungbluth, Goetze & Lenz, 2013), and no prior deep-sea plankton studies have attempted taxonomic assignment of nauplii (e.g., Christiansen et al., 1999; Wishner, 1980; Kersten, Smith & Vetter, 2017). We combined a 63 µm mesh size for collections with metabarcoding analyses, enabling us to detect this highly diverse and almost entirely unknown component of the meroplanktonic fauna. Copepods are also a central component of the deep-sea meiobenthos in the CCZ and abyssal Pacific (Ahnert & Schriever, 2001; Radziejewska, 2002), and we also sampled several representatives from this assemblage, including the genera *Pontostratiotes* and *Parameiropsis,* and the families Schminkepinellidae, Smirnovipinidae and Ectinosomatidae (Table 2).

Several mechanisms may generate spatial patchiness in the larval assemblage, as was observed in site-to-site variability of larval diversity and community structure. Our results indicated low spatial structuring over distances of 100 km (between UK1 and OMS1 mining claim areas), but substantial variability in the larval assemblage within distances of 5–50 km, likely in part due to under-sampling (Figs. 5, 6, S1). The diversity and structure of the BBL larval assemblage is dependent on the community structure and reproductive output of the parental populations, larval type (lecithotrophic, planktotrophic), vertical positioning of larvae above the seafloor, larval mortality, and pelagic larval duration (PLD) (e.g., Cowen & Sponaugle, 2009; Mullineaux et al., 2012; Mullineaux et al., 2013; Mills et al., 2013). Adults of most of the meroplankton classes detected in this study have been previously reported in the CCZ (Wilson, 2017; De Smet et al., 2017), and similar spatial patterns may occur for both adult populations and larval assemblages. In the CCZ, adult macrofaunal communities have been shown to be distinct on a scale of 1,000 km (Janssen et al., 2015; Glover et al., 2002), most likely driven by large-scale gradients in POM flux at the seafloor (Smith et al., 2008a). However, it remains unclear to what degree the CCZ fauna is variable at regional or smaller spatial scales, as under-sampling remains an issue. For mega- to meiofanual communities, differences in seafloor POC flux, nodule cover and sediment characteristics among sites may contribute to spatial differences in abundance and diversity (Amon et al., 2016; De Smet et al., 2017; Simon-Lledó et al., 2019). Polymetallic nodules provide (micro-)habitat heterogeneity and create additional niches for a specialized nodule fauna (e.g., Amon et al., 2016; Vanreusel et al., 2016; Veillette et al., 2007), with abundance, community composition, and distribution of CCZ mega-, macro- and meiofauna partially structured by the presence of nodules (e.g., Simon-Lledó et al., 2019; Smith et al., 2008a; Vanreusel et al., 2016; Veillette et al., 2007). Nodule cover and megafaunal community structure have been observed to differ significantly within distances of 5–50 km (between sampling sites) (Amon et al., 2016; Simon-Lledó et al., 2019), and it is possible that larval patchiness is caused by differential contributions to the meroplankton from localized and distinctively structured benthic communities. Finally, the mesoscale patchiness of the larval assemblage could be driven in part by short pelagic larval durations (PDL) and short dispersal distances for some members of the assemblage. Although dispersal distances of deep-sea taxa are largely unknown, especially for abyssal regions, prior studies report deep-sea invertebrates having a pelagic larval duration (PLD) of ∼10–300 days (Hilário et al., 2015; Baco et al., 2016), yielding an estimated dispersal distance of several kilometers to several hundred kilometers in the abyssal CCZ (current meter data in Table S1; Baco et al., 2016). The mesoscale patchiness we observed on a scale of ∼20 days and across 5–50 km, as well as the high diversity of taxa sampled within the benthic boundary layer, suggest that many CCZ taxa may be dispersing close to the bottom, with PLD and dispersal distances at the low end of this estimated dispersal range. If high larval patchiness and limited dispersal ranges are the rule in the abyssal CCZ (excluding a few taxa that might disperse higher in the water column), nodule mining could increase spatial fragmentation of adult benthic populations by removing larval source populations over substantial areas (hundreds to thousands of square kilometers; Smith et al., 2008b; Simon-Lledó et al., 2019). Temporal variation of the larval assemblage in the CCZ on the scale of days is likely negligible due to little seasonality of POC flux at the seafloor in the mesotrophic abyss (Hannides & Smith, 2003) and stable abiotic factors throughout the study area (Table S1).

Integrated morphological and molecular approaches yield far more information about larval diversity, abundance, and temporal and spatial variability than either approach alone; thus, we recommend such an integrated approach for future research on larval assemblages in the deep ocean. Notably, even though all visible pre-adult stages were removed from the samples, metabarcoding captured much higher meroplankton diversity than microscopy-based methods. As a large fraction of the crustacean larvae in the form of nauplii were not removed from the metabarcoding fraction due to the inability to assign taxonomy to these life stages, inclusion of these larval specimens likely explains the majority of detected DNA not contained in individually barcoded larvae (as opposed to environmental DNA). For the remaining taxa, we speculate that DNA may have been present in small, damaged, or unidentifiable specimens of soft-bodied, more fragile groups (e.g., partial specimens, particulate material in the BBL). Given highly diverse deep-sea communities, it is also important to use multiple markers in metabarcoding studies in order to capture the entirety of the larval assemblage. In this study, only ca. 3% of meroplankton taxa classified to the family level were identified by all three markers and the great majority were detected by only one (Tables 2, 3). Success in taxonomic assignment also varies across markers; for example, the mtCOI amplicon enabled greater resolution than 18S, with classification of more than 19% of the captured meroplanktonic taxa (Tables 2, 3). Taxonomic assignment of metabarcoding OTUs requires a comprehensive reference sequence database that links the original taxonomic descriptions of species with sequence data (Deagle et al., 2014; Bucklin et al., 2016; Sinniger et al., 2016). Yet publicly available reference sequences do not exist for the majority of deep-sea species (Sinniger et al., 2016), in part because many species remain undescribed. For example, there are currently no records in NCBI for the cyclopoid genus *Barathricola* or the harpacticoid family Cerviniidae, both of which were common in our samples, as identified by microscopy. In order to increase the taxonomic resolution and power of metabarcoding methods for deep sea studies, it is imperative to continue to acquire reference sequences by combining traditional morphological taxonomy with molecular approaches, thereby continuing to build reference databases (e.g., Janssen et al., 2015; Glover et al., 2015; Glover et al., 2018).

## Conclusions

Knowledge of the diversity, composition and spatial distribution of abyssal near-bottom meroplankton assemblages is fundamental to understanding the ecology of benthic invertebrate populations of the abyssal plain, including the processes of dispersal, recruitment and recovery of abyssal populations potentially impacted by polymetallic nodule mining (Smith et al., 2008a; Wedding et al., 2015; Baco et al., 2016). The metabarcoding approach used here provided new insights into the near-bottom meroplankton assemblage in the eastern CCZ, a region of particular interest for deep-sea polymetallic nodule mining. Primary observations from this study are that (1) the meroplankton of the CCZ benthic boundary layer is a diverse assemblage with representatives from 12 Phyla, 23 Classes, 46 Orders and 65 Families; (2) the assemblage includes a group of parasitic and commensal organisms that were previously unknown or highly under-sampled in the deep ocean, in addition to commonly reported larval groups (e.g., bivalves, polychaetes, gastropods) and several representatives of chemosynthetic deep ocean habitats; (3) larvae are spatially patchy at the mesoscale (5–50 km) across the abyssal plain, though larger volume samples are required to fully sample the larval diversity within each claim area; and (4) metabarcoding and larval DNA barcoding of individual larvae combined detected a 2.7–4.3 times higher diversity than morphology-based analyses of these same quantitative samples. Our results therefore significantly advance our knowledge of the poorly studied larval assemblages over the abyssal plains in the Pacific Ocean, and provide key baseline data on larval pools and potential connectivity of benthic populations likely to be impacted by polymetallic nodule mining in the CCZ.

##  Supplemental Information

10.7717/peerj.7691/supp-1 Table S1 Station overview of environmental, metabarcoding, barcoding, and morphological data Time = Local time at pumping start. Depth = Bottom depth. Current and temperature data were collected by Leitner et al. (2017) 1.5 m above the seafloor approximately 1–3 km away from sampling sites. Number of sequences is reported after all quality filtering steps and prior to subsampling. OTU numbers are reported after subsampling. The DNA barcoding section reports combined results from the same three markers used for metabarcoding. H′= Shannon-Weaver index. D = Simpson Diversity index. J = Pielou’s evenness index.

10.7717/peerj.7691/supp-2 Table S2 Primer sets used for (A) metabarcoding of plankton pump samples, and (B) DNA barcoding of individual larvae collected in the abyssal CCZ References are listed in File S2.

10.7717/peerj.7691/supp-3 Table S3 Overview of raw sequencing and metadata files used in this study and available in public repositories

10.7717/peerj.7691/supp-4 Table S4 Statistical tests regarding spatial variability in the meroplankton assemblage PERMANOVA with distance matrix, ANOSIM, MRPP, and Mantel tests were performed for each marker with 999 permutations for the presence/absence and non-transformed (in parentheses) datasets. Multivariate homogeneity of groups dispersions was tested with the betadisper and anova functions in R (999 permutations) to assess the validity of the ANOSIM tests. The indeces of species richness, evenness, and diversity are mean values and significance was determined with t-tests. S(obs) = No. of OTUs observed. H′= Shannon-Weaver index. D = Simpson Diversity index. J = Pielou’s evenness index.

10.7717/peerj.7691/supp-5 Table S5 Barcoding results for abyssal larvae using three fragments of the 18S rRNA and mtCOI genes Specimens were taxonomically classified via a consensus call using the SILVA database, NCBI Genbank and the Statistical Assignment Package. Metabarcoding OTUs from each marker were classified applying the same methods to representative sequences for each OTU. *No bivalve OTUs in the 18S_V1&2 metabarcoding dataset. ** a sipunculid.

10.7717/peerj.7691/supp-6 Table S6 Meroplankton OTUs identified by microscopy representing an extended dataset of Kersten, Smith & Vetter (2017) Information on the ecology or existing sample records are included for taxa classified to the family level and beyond. References are listed in File S2.

10.7717/peerj.7691/supp-7 Figure S1 Community richness estimators compared between two sampling strata for three metabarcoding markers (A) Jackknife 1, (B) Bootstrap and (C) Species accumulation curves compared between markers and sampling strata. All plots include both barcoding and metabarcoding data.

10.7717/peerj.7691/supp-8 Figure S2 Distribution of operational taxonomic units (OTUs) across meroplanktonic groups compared for three metabarcoding markers (A) proportion of OTUs (%), and (B) % sequence reads are shown for 10 dominant taxonomic groups with meroplanktonic representatives. Nematoda (0.7% of all meroplankton OTUs combining the three metabarcoding datasets), Tantulocarida (0.7%), Tunicata (2.2%), Vertebrata (3.6%), Chitonida (0.7%), Xenacoelomorpha (0.4%), Decapoda (0.4%), Pedunculata (1.1%), Pycnogonida (0.4%), Entoprocta (1.4%), Nemertea (0.7%), Scaphopoda (0.4%), and Sipuncula (0.4%) were grouped into Others (12.9% total). Labels for 18S markers V1&2 and V7&8 are abbreviated.

10.7717/peerj.7691/supp-9 Figure S3 Venn diagrams depicting taxonomic overlap in meroplankton diversity recovered using three metabarcoding markers Numbers in parentheses represent the total number of meroplankton families (A) and genera (B) captured by each marker. The number of families is larger than the number of genera, due to many OTUs being classified only to family.

10.7717/peerj.7691/supp-10 Figure S4 Spatial patchiness and structure of the meroplankton assemblage captured by two metabarcoding markers across 12 abyssal sites The non-parametric multi-dimensional scaling (NMDS) plot (A) and cluster dendrogram (B) were constructed for the 18S_V7&8 and mtCOI marker. The NMDS plot show the placement of 12 abyssal sites (UK1 + OMS1) in ordination space, colored by strata. The dendrogram is based on a hierarchical cluster analysis using group average linkage and the Bray-Curtis distance measure. OTU sequence abundance was transformed into presence/absence prior to both analyses.

10.7717/peerj.7691/supp-11 Figure S5 Compositional difference of the meroplankton assemblage between the UK and OMS stratum detected by three metabarcoding markers Proportion of operational taxonomic units (% OTUs) and % sequence reads are shown for the four most abundant taxa captured by (A) 18S_V1&2, (B) 18S_V7&8, and (C) mtCOI. Error bars show standard error. Nematoda (0.7% of all meroplankton OTUs combining the three metabarcoding datasets), Tantulocarida (0.7%), Tunicata (2.2%), Vertebrata (3.6%), Chitonida (0.7%), Xenacoelomorpha (0.4%), Decapoda (0.4%), Pedunculata (1.1%), Pycnogonida (0.4%), Entoprocta (1.4%), Nemertea (0.7%), Scaphopoda (0.4%), and Sipuncula (0.4%) were grouped into Others (12.9% total).

10.7717/peerj.7691/supp-12 File S1 Supplemental Material and Methods

10.7717/peerj.7691/supp-13 Supplemental Information 1 Table References

## Additional Information and Declarations

### Data Availability

The following information was supplied regarding data availability: Data are available at the Dryad Digital Repository, including OTU tables with taxonomic identification and presence/absence at sampling stations for each OTU and marker, and fasta files with representative sequences for each OTU and marker: doi: 10.5061/dryad.vb68g9d. Unique, non-chimeric, FASTQ sequence files for each marker used in this study are available under the NCBI BioProject: PRJNA548951 and the NCBI Sequence Read Archive (SRA): SRR9304915, SRR9304914, and SRR9304913.

## References

[ref-1] Adams DK, Arellano SM, Govenar B (2012). Larval dispersal: vent life in the water column. Oceanography.

[ref-2] Ahnert A, Schriever G (2001). Response of abyssal Copepoda Harpacticoida (Crustacea) and other meiobenthos to an artificial disturbance and its bearing on future mining for polymetallic nodules. Deep Sea Research Part II: Topical Studies in Oceanography.

[ref-3] Amon DJ, Hilario A, Arbizu PM, Smith CR (2017a). Observations of organic falls from the abyssal Clarion-Clipperton Zone in the tropical eastern Pacific Ocean. Marine Biodiversity.

[ref-4] Amon DJ, Ziegler AF, Dahlgren TG, Glover AG, Goineau A, Gooday AJ, Wiklund H, Smith CR (2016). Insights into the abundance and diversity of abyssal megafauna in a polymetallic-nodule region in the eastern Clarion-Clipperton Zone. Scientific Reports.

[ref-5] Amon DJ, Ziegler AF, Drazen JC, Grischenko AV, Leitner AB, Lindsay DJ, Voight JR, Wicksten MK, Young CM, Smith CR (2017b). Megafauna of the UKSRL exploration contract area and eastern Clarion-Clipperton Zone in the Pacific Ocean: annelida, Arthropoda, Bryozoa, Chordata, Ctenophora, Mollusca. Biodiversity Data Journal.

[ref-6] Amon DJ, Ziegler AF, Kremenetskaia A, Mah CL, Mooi R, O’Hara T, Pawson DL, Roux M, Smith CR (2017c). Megafauna of the UKSRL exploration contract area and eastern Clarion-Clipperton Zone in the Pacific Ocean: echinodermata. Biodiversity Data Journal.

[ref-7] Anderson MJ (2001). A new method for non-parametric multivariate analysis of variance. Austral Ecology.

[ref-8] Anderson MJ (2006). Distance-based tests for homogeneity of multivariate dispersions. Biometrics.

[ref-9] Baco AR, Etter RJ, Ribeiro PA, Der Heyden S, Beerli P, Kinlan BP (2016). A synthesis of genetic connectivity in deep-sea fauna and implications for marine reserve design. Molecular Ecology.

[ref-10] Beaulieu SE, Mullineaux LS, Adams DK, Mills SW (2009). Comparison of a sediment trap and plankton pump for timeseries sampling of larvae near deep-sea hydrothermal vents. Limnology and Oceanography: Methods.

[ref-11] Boxshall GA (1998). Host specificity in copepod parasites of deep-sea fishes. Journal of Marine Systems.

[ref-12] Boxshall GA, Halsey SH (2004). An introduction to copepod diversity.

[ref-13] Bucklin A, Lindeque PK, Rodriguez-Ezpeleta N, Albaina A, Lehtiniemi M (2016). Metabarcoding of marine zooplankton: prospects, progress and pitfalls. Journal of Plankton Research.

[ref-14] Cho DH, Wi JH, Suh H-L (2016). Two new species of the deep-sea genus *Parameiropsis* (Copepoda: Harpacticoida) from the eastern central Pacific. Zootaxa.

[ref-15] Christiansen B, Drüke B, Koppelmann R, Weikert H (1999). The near-bottom zooplankton at the abyssal BIOTRANS site. Journal of Plankton Research.

[ref-16] Clarke KR (1993). Non-parametric multivariate analyses of changes in community structure. Austral Ecology.

[ref-17] Cowen RK, Sponaugle S (2009). Larval dispersal and marine population connectivity. Annual Review of Marine Science.

[ref-18] Dahlgren TG, Wiklund H, Rabone M, Amon DJ, Ikebe C, Watling L, Smith CR, Glover AG (2016). Abyssal fauna of the UK-1 polymetallic nodule exploration area, Clarion-Clipperton Zone, central Pacific Ocean: Cnidaria. Biodiversity Data Journal.

[ref-19] De Buron I, Morand S (2004). Deep-sea hydrothermal vent parasites: why do we not find more?. Parasitology.

[ref-20] De Smet B, Pape E, Riehl T, Bonifácio P, Colson L, Vanreusel A (2017). The community structure of deep-sea macrofauna associated with polymetallic nodules in the eastern part of the clarion-clipperton fracture zone. Frontiers in Marine Science.

[ref-21] Deagle BE, Clarke LJ, Kitchener JA, Polanowski AM, Davidson AT (2018). Genetic monitoring of open ocean biodiversity: an evaluation of DNA metabarcoding for processing continuous plankton recorder samples. Molecular Ecology Resources.

[ref-22] Deagle BE, Jarman SN, Coissac E, Pompanon F, Taberlet P (2014). DNA metabarcoding and the cytochrome c oxidase subunit I marker: not a perfect match. Biology Letters.

[ref-23] Deagle BE, Thomas AC, Shaffer AK, Trites AW, Jarman SN (2013). Quantifying sequence proportions in a DNA-based diet study using Ion Torrent amplicon sequencing: which counts count?. Molecular Ecology Resources.

[ref-24] Djurhuus A, Pitz K, Sawaya NA, Rojas-Márquez J, Michaud B, Montes E, Muller-Karger F, Breitbart M (2018). Evaluation of marine zooplankton community structure through environmental DNA metabarcoding: metabarcoding zooplankton from eDNA. Limnology and Oceanography: Methods.

[ref-25] Durden JM, Bett BJ, Jones DOB, Huvenne VAI, Ruhl HA (2015). Abyssal hills—hidden source of increased habitat heterogeneity, benthic megafaunal biomass and diversity in the deep sea. Progress in Oceanography.

[ref-26] Fonseca VG, Carvalho GR, Sung W, Johnson HF, Power DM, Neill SP, Packer M, Blaxter ML, Lambshead PJD, Thomas WK (2010). Second-generation environmental sequencing unmasks marine metazoan biodiversity. Nature Communications.

[ref-27] Glover A, Dahlgren T, Wiklund H, Mohrbeck I, Smith C (2015). An end-to-end DNA taxonomy methodology for benthic biodiversity survey in the clarion-clipperton zone, central pacific abyss. Journal of Marine Science and Engineering.

[ref-28] Glover AG, Smith CR (2003). The deep-sea floor ecosystem- current status and prospects of anthropogenic change by the year 2025. Environmental Conservation.

[ref-29] Glover AG, Smith CR, Paterson G, Wilson G, Hawkins L, Sheader M (2002). Polychaete species diversity in the central Pacific abyss: local and regional patterns, and relationships with productivity. Marine Ecology Progress Series.

[ref-30] Glover AG, Wiklund H, Chen C, Dahlgren TG (2018). Managing a sustainable deep-sea ‘blue economy’ requires knowledge of what actually lives there. eLife.

[ref-31] Glover AG, Wiklund H, Rabone M, Amon DJ, Smith CR, O’Hara T, Mah CL, Dahlgren TG (2016). Abyssal fauna of the UK-1 polymetallic nodule exploration claim, Clarion-Clipperton Zone, central Pacific Ocean: Echinodermata. Biodiversity Data Journal.

[ref-32] Goineau A, Gooday AJ (2017). Novel benthic foraminifera are abundant and diverse in an area of the abyssal equatorial Pacific licensed for polymetallic nodule exploration. Scientific Reports.

[ref-33] Good IJ (1953). The population frequencies of species and the estimation of population parameters. Biometrika.

[ref-34] Gotelli NJ, Colwell RK (2001). Quantifying biodiversity: procedures and pitfalls in the measurement and comparison of species richness. Ecology Letters.

[ref-35] Hannides AK, Smith CR, Black KD, Shimmield GD (2003). The northeastern pacific abyssal plain. Biogeochemistry of marine systems.

[ref-36] Harris PT, Macmillan-Lawler M, Rupp J, Baker EK (2014). Geomorphology of the oceans. Marine Geology.

[ref-37] Heberle H, Meirelles GV, Da Silva FR, Telles GP, Minghim R (2015). InteractiVenn: a web-based tool for the analysis of sets through Venn diagrams. BMC Bioinformatics.

[ref-38] Hein J, Mizell K, Koschinsky A, Conrad TA (2013). Deep-ocean mineral deposits as a source of critical metals for high- and green-technology applications: comparison with land-based resources. Ore Geology Reviews.

[ref-39] Hilário A, Metaxas A, Gaudron SM, Howell KL, Mercier A, Mestre NC, Ross RE, Thurnherr AM, Young C (2015). Estimating dispersal distance in the deep sea: challenges and applications to marine reserves. Frontiers in Marine Science.

[ref-40] Hirai J, Katakura S, Kasai H, Nagai S (2017). Cryptic zooplankton diversity revealed by a metagenetic approach to monitoring metazoan communities in the coastal waters of the okhotsk sea, Northeastern Hokkaido. Frontiers in Marine Science.

[ref-41] Huber JA, Welch DBM, Morrison HG, Huse SM, Neal PR, Butterfield DA, Sogin ML (2007). Microbial population structures in the deep marine biosphere. Science.

[ref-42] Hutchings P (1998). Biodiversity and functioning of polychaetes in benthic sediments. Biodiversity & Conservation.

[ref-43] ISA (2010). A geological model of the polymetallic nodule deposits in the Clarion-Clipperton Fracture Zone. ISA_Technical Study No. 6.

[ref-44] Janssen A, Kaiser S, Meißner K, Brenke N, Menot L, Arbizu PM (2015). A reverse taxonomic approach to assess macrofaunal distribution patterns in abyssal Pacific polymetallic nodule fields. PLOS ONE.

[ref-45] Jumars PA, Dorgan KM, Lindsay SM (2015). Diet of worms emended: an update of polychaete feeding guilds. Annual Review of Marine Science.

[ref-46] Jungbluth MJ, Goetze E, Lenz P (2013). Measuring copepod naupliar abundance in a subtropical bay using quantitative PCR. Marine Biology.

[ref-47] Kaiser S, Smith CR, Arbizu PM (2017). Editorial: biodiversity of the clarion clipperton fracture zone. Marine Biodiversity.

[ref-48] Kersten O (2015). Abyssal near-bottom zooplankton in the Eastern Tropical North Pacific. Unpublished master’s thesis.

[ref-49] Kersten O, Smith CR, Vetter EW (2017). Abyssal near-bottom dispersal stages of benthic invertebrates in the Clarion-Clipperton polymetallic nodule province. Deep Sea Research Part I: Oceanographic Research Papers.

[ref-50] Kozich JJ, Westcott SL, Baxter NT, Highlander SK, Schloss PD (2013). Development of a dual-index sequencing strategy and curation pipeline for analyzing amplicon sequence data on the MiSeq Illumina sequencing platform. Applied and Environmental Microbiology.

[ref-51] Kruskal JB, Wish M (1978). Multidimensional scaling.

[ref-52] Leitner AB, Neuheimer AB, Donlon E, Smith CR, Drazen JC (2017). Environmental and bathymetric influences on abyssal bait-attending communities of the Clarion Clipperton Zone. Deep Sea Research Part I: Oceanographic Research Papers.

[ref-53] Leray M, Knowlton N (2015). DNA barcoding and metabarcoding of standardized samples reveal patterns of marine benthic diversity. Proceedings of the National Academy of Sciences of the United States of America.

[ref-54] Leray M, Yang JY, Meyer CP, Mills SC, Agudelo N, Ranwez V, Boehm JT, Machida RJ (2013). A new versatile primer set targeting a short fragment of the mitochondrial COI region for metabarcoding metazoan diversity: application for characterizing coral reef fish gut contents. Frontiers in Zoology.

[ref-55] Letunic I (2018). ‘phyloT: a phylogenetic tree generator’. https://phylot.biobyte.de/.

[ref-56] Letunic I, Bork P (2016). Interactive tree of life (iTOL) v3: an online tool for the display and annotation of phylogenetic and other trees. Nucleic Acids Research.

[ref-57] Lindeque PK, Parry HE, Harmer RA, Somerfield PJ, Atkinson A (2013). Next generation sequencing reveals the hidden diversity of zooplankton assemblages. PLOS ONE.

[ref-58] Machida RJ, Knowlton N (2012). PCR primers for metazoan nuclear 18S and 28S ribosomal DNA sequences. PLOS ONE.

[ref-59] Machida RJ, Leray M, Ho S-L, Knowlton N (2017). Metazoan mitochondrial gene sequence reference datasets for taxonomic assignment of environmental samples. Scientific Data.

[ref-60] Magurran AE (2004). Measuring biological diversity.

[ref-61] Mantel N (1967). The detection of disease clustering and a generalized regression approach. Cancer Research.

[ref-62] McClain CR, Hardy SM (2010). The dynamics of biogeographic ranges in the deep sea. Proceedings of the Royal Society B.

[ref-63] Metaxas A (2011). Spatial patterns of larval abundance at hydrothermal vents on seamounts: evidence for recruitment limitation. Marine Ecology Progress Series.

[ref-64] Mielke PW, Berry KJ, Johnson ES (1976). Multi-response permutation procedures for *a priori* classifications. Communications in Statistics - Theory and Methods.

[ref-65] Mills SW, Mullineaux LS, Beaulieu SE, Adams DK (2013). Persistent effects of disturbance on larval patterns in the plankton after an eruption on the East Pacific Rise. Marine Ecology Progress Series.

[ref-66] Mullineaux LS, Adams DK, Mills SW, Beaulieu SE (2010). Larvae from afar colonize deep-sea hydrothermal vents after a catastrophic eruption. Proceedings of the National Academy of Sciences of the United States of America.

[ref-67] Mullineaux LS, Le Bris N, Mills SW, Henri P, Bayer SR, Secrist RG, Siu N (2012). Detecting the influence of initial pioneers on succession at deep-sea vents. PLOS ONE.

[ref-68] Mullineaux LS, McGillicuddy DJ, Mills SW, Kosnyrev VK, Thurnherr AM, Ledwell JR, Lavelle JW (2013). Active positioning of vent larvae at a mid-ocean ridge. Deep Sea Research Part II: Topical Studies in Oceanography.

[ref-69] Mullineaux LS, Mills SW, Sweetman AK, Beaudreau AH, Metaxas A, Hunt HL (2005). Vertical, lateral and temporal structure in larval distributions at hydrothermal vents. Marine Ecology Progress Series.

[ref-70] Munch K, Boomsma W, Huelsenbeck JP, Willerslev E, Nielsen R (2008). Statistical assignment of DNA sequences using Bayesian phylogenetics. Systematic Biology.

[ref-71] Oksanen J, Blanchet FG, Friendly M, Kindt R, Legendre P, McGlinn D, Minchin PR, O’Hara RB, Simpson GL, Solymos P, Stevens MHH, Szoecs E, Wagner H (2018). https://CRAN.R-project.org/package=vegan.

[ref-72] Pielou EC (1966). The measurement of diversity in different types of biological collections. Journal of Theoretical Biology.

[ref-73] Quast C, Pruesse E, Yilmaz P, Gerken J, Schweer T, Yarza P, Peplies J, Glöckner FO (2013). The SILVA ribosomal RNA gene database project: improved data processing and web-based tools. Nucleic Acids Research.

[ref-74] R Core Team (2018). https://www.R-project.org/.

[ref-75] Radziejewska T (2002). Responses of deep-sea meiobenthic communities to sediment disturbance simulating effects of polymetallic nodule mining. International Review of Hydrobiology.

[ref-76] Ramirez-Llodra E, Brandt A, Danovaro R, Escobar E, German CR, Levin LA, Arbizu PM, Menot L, Buhl-Mortensen P, Narayanaswamy BE (2010). Deep, diverse and definitely different: unique attributes of the world’s largest ecosystem. Biogeosciences.

[ref-77] Ranwez V, Harispe S, Delsuc F, Douzery EJP (2011). MACSE: multiple alignment of coding sequences accounting for frameshifts and stop codons. PLOS ONE.

[ref-78] Rognes T, Flouri T, Nichols B, Quince C, Mahé F (2016). VSEARCH: a versatile open source tool for metagenomics. PeerJ.

[ref-79] Schloss PD, Westcott SL, Ryabin T, Hall JR, Hartmann M, Hollister EB, Lesniewski RA, Oakley BB, Parks DH, Robinson CJ (2009). Introducing mothur: open-source, platform-independent, community-supported software for describing and comparing microbial communities. Applied and Environmental Microbiology.

[ref-80] Shannon CE, Weaver W (1948). The mathematical theory of communication.

[ref-81] Shulse CN, Maillot B, Smith CR, Church MJ (2017). Polymetallic nodules, sediments, and deep waters in the equatorial North Pacific exhibit highly diverse and distinct bacterial, archaeal, and microeukaryotic communities. MicrobiologyOpen.

[ref-82] Simon-Lledó E, Schoening T, Benoist NMA, Bett BJ, Huvenne VAI, Jones DOB (2019). Ecology of a polymetallic nodule occurrence gradient: implications for deep-sea mining. Limnology and Oceanography.

[ref-83] Simpson EH (1949). Measurement of diversity. Nature.

[ref-84] Sinniger F, Pawlowski J, Harii S, Gooday AJ, Yamamoto H, Chevaldonné P, Cedhagen T, Carvalho G, Creer S (2016). Worldwide analysis of sedimentary dna reveals major gaps in taxonomic knowledge of deep-sea benthos. Frontiers in Marine Science.

[ref-85] Smith CR, De Leo FC, Bernardino AF, Sweetman AK, Arbizu PM (2008a). Abyssal food limitation, ecosystem structure and climate change. Trends in Ecology & Evolution.

[ref-86] Smith CR, Glover AG, Treude T, Higgs ND, Amon DJ (2015). Whale-fall ecosystems: recent insights into ecology, paleoecology, and evolution. Annual Review of Marine Science.

[ref-87] Smith CR, Levin LA, Koslow A, Tyler PA, Polunin N (2008b). The near future of the deep seafloor ecosystems. In Aquatic ecosystems : trends and global prospects.

[ref-88] Sogin ML, Morrison HG, Huber JA, Welch DM, Huse SM, Neal PR, Arrieta JM, Herndl GJ (2006). Microbial diversity in the deep sea and the underexplored ‘rare biosphere’. Proceedings of the National Academy of Sciences of the United States of America.

[ref-89] Sommer SA, Van Woudenberg L, Lenz PH, Cepeda G, Goetze E (2017). Vertical gradients in species richness and community composition across the twilight zone in the North Pacific Subtropical Gyre. Molecular Ecology.

[ref-90] Sweetman AK, Smith CR, Shulse CN, Maillot B, Lindh M, Church MJ, Meyer KS, Van Oevelen D, Stratmann T, Gooday AJ (2018). Key role of bacteria in the short-term cycling of carbon at the abyssal seafloor in a low particulate organic carbon flux region of the eastern Pacific Ocean: key role of benthic bacteria in abyssal food webs. Limnology and Oceanography.

[ref-91] Taboada S, Riesgo A, Wiklund H, Paterson GLJ, Koutsouveli V, Santodomingo N, Dale AC, Smith CR, Jones DOB, Dahlgren TG, Glover AG (2018). Implications of population connectivity studies for the design of marine protected areas in the deep sea: an example of a demosponge from the Clarion-Clipperton Zone. Molecular Ecology.

[ref-92] Vanreusel A, Hilario A, Ribeiro PA, Menot L, Arbizu PM (2016). Threatened by mining, polymetallic nodules are required to preserve abyssal epifauna. Scientific Reports.

[ref-93] Veillette J, Juniper SK, Gooday AJ, Sarrazin J (2007). Influence of surface texture and microhabitat heterogeneity in structuring nodule faunal communities. Deep Sea Research Part I: Oceanographic Research Papers.

[ref-94] Vinogradova NG, Southward AJ, Gebruk AV, Southward EC, Tyler PA (1997). Zoogeography of the abyssal and hadal zones. In the biogeography of the oceans, advances in marine biology.

[ref-95] Vrijenhoek RC (2010). Genetic diversity and connectivity of deep-sea hydrothermal vent metapopulations. Molecular Ecology.

[ref-96] Wang Q, Garrity GM, Tiedje JM, Cole JR (2007). Naive Bayesian classifier for rapid assignment of rRNA sequences into the new bacterial taxonomy. Applied and Environmental Microbiology.

[ref-97] Wedding LM, Friedlander AM, Kittinger JN, Watling L, Gaines SD, Bennett M, Hardy SM, Smith CR (2013). From principles to practice: a spatial approach to systematic conservation planning in the deep sea. Proceedings of Biological Sciences / The Royal Society.

[ref-98] Wedding LM, Reiter SM, Smith CR, Gjerde KM, Kittinger JN, Friedlander AM, Gaines SD, Clark MR, Thurnherr AM, Hardy SM (2015). Managing mining of the deep seabed. Science.

[ref-99] Wiklund H, Taylor JD, Dahlgren TG, Todt C, Ikebe C, Rabone M, Glover AG (2017). Abyssal fauna of the UK-1 polymetallic nodule exploration area, Clarion-Clipperton Zone, central Pacific Ocean: Mollusca. ZooKeys.

[ref-100] Wilson GDF (2017). Macrofauna abundance, species diversity and turnover at three sites in the Clipperton-Clarion Fracture Zone. Marine Biodiversity.

[ref-101] Wishner KF (1980). Aspects of the community ecology of deep-sea, benthopelagic plankton, with special attention to gymnopleid copepods. Marine Biology.

[ref-102] Yilmaz P, Parfrey LW, Yarza P, Gerken J, Pruesse E, Quast C, Schweer T, Peplies J, Ludwig W, Glöckner FO (2014). The SILVA and ‘All-species Living Tree Project (LTP)’ taxonomic frameworks. Nucleic Acids Research.

[ref-103] Young CM, He R, Emlet RB, Li Y, Qian H, Arellano SM, Van Gaest A, Bennett KC, Wolf M, Smart TI, Rice ME (2012). Dispersal of deep-sea larvae from the intra-American seas: simulations of trajectories using ocean models. Integrative and Comparative Biology.

[ref-104] Zhang J, Kobert K, Flouri T, Stamatakis A (2014). PEAR: a fast and accurate Illumina Paired-End reAd mergeR. Bioinformatics.

