# Supplementary material for: Larval assemblages over the abyssal plain in the Pacific are highly diverse and spatially patchy
Source: PeerJ. 2019 Sep 26;7:e7691. doi: 10.7717/peerj.7691 (PMC6766376; doi:10.7717/peerj.7691)
Supplement: Table S1 — Time = Local time at pumping start. Depth = Bottom depth. Current and temperature data were collected by Leitner et al. (2017) 1.5 m above the seafloor approximately 1–3 km away from sampling sites. Number of sequences is reported after all quality filtering steps and prior to subsampling. OTU numbers are reported after subsampling. The DNA barcoding section reports combined results from the same three markers used for metabarcoding. H′= Shannon-Weaver index. D = Simpson Diversity index. J = Pielou’s evenness index. [file peerj-07-7691-s001.pdf]

		Station #	1	2	6	7	8	9	3	4	5	10	11	12
Environmental Parameters		Stratum	UK1						OMS1					
		Latitutde	12°23.098' N	12°33.820' N	12°30.224' N	12°33.674' N	12°26.158' N	12°26.060' N	12°07.071' N	12°03.932' N	12°11.526' N	12°08.671' N	12°03.282' N	12°01.658' N
		Longitude	116° 28.151' W	116° 44.112' W	116° 37.310' W	116° 32.379' W	116° 37.872' W	116° 35.822' W	117° 22.202' W	117° 10.049' W	117° 18.930' W	117° 13.951' W	117° 14.082' W	117° 20.490' W
		Time	18:08	15:08	17:12	14:50	12:50	14:42	9:45	9:56	10:55	9:45	6:59	12:22
		Depth (m)	4128	4176	4220	4237	4142	4132	4144	4111	4114	4082	4076	4172
		Average Current Speed (m/s)	0.09	0.06	0.09	0.04	0.06	0.04	0.06	0.04	0.04	0.04	0.04	0.04
		Maximum Current Speed (m/s)	0.2	0.23	0.27	0.12	0.15	0.15	0.18	0.17	0.13	0.18	0.15	0.12
		Temperature (°C)	1.52	1.83	1.58	1.59	1.58	1.58	1.57	1.56	1.56	1.56	1.56	1.56
		Seawater Volume Filtered (m3)	59.43	62.40	35.46	34.08	35.51	34.77	34.72	34.92	34.29	35.42	35.25	34.92
Metabarcoding	18S_V1&2	# of Sequences (N)	8322	7321	12400	18559	18763	7625	6423	3681	8220	5842	7298	5400
		Good's Coverage	0.984	0.981	0.985	0.983	0.984	0.985	0.982	0.986	0.985	0.984	0.985	0.983
		# of Meroplankton OTUs (N)	18	23	23	26	15	17	16	5	22	18	10	9
		J	0.49	0.59	0.36	0.87	0.54	0.84	0.64	0.52	0.80	0.52	0.78	0.82
		H'	1.42	1.85	1.14	2.85	1.47	2.38	1.79	0.84	2.47	1.49	1.79	1.80
		D	0.52	0.73	0.42	0.92	0.65	0.88	0.75	0.40	0.87	0.57	0.81	0.79
	18S_V7&8	# of Sequences (N)	3729	5404	2398	3495	1897	2380	1857	1229	2772	1926	1872	4625
		Good's Coverage	0.821	0.830	0.843	0.832	0.853	0.835	0.824	0.866	0.799	0.857	0.836	0.860
		# of Meroplankton OTUs (N)	11	33	20	33	24	29	27	10	26	26	22	14
		J	0.84	0.85	0.74	0.83	0.74	0.82	0.83	0.81	0.84	0.62	0.87	0.83
		H'	2.01	2.99	2.22	2.89	2.35	2.76	2.72	1.85	2.74	2.02	2.68	2.20
		D	0.82	0.92	0.81	0.91	0.82	0.90	0.91	0.79	0.91	0.76	0.91	0.86
	mtCOI	# of Sequences (N)	15045	8313	1801	3623	22959	8397	1288	1733	11152	4795	8306	7566
		Good's Coverage	0.963	0.962	0.978	0.978	0.966	0.977	0.967	0.974	0.970	0.982	0.978	0.976
		# of Meroplankton OTUs (N)	15	18	10	13	10	14	16	5	12	8	9	9
		J	0.84	0.58	0.75	0.62	0.68	0.69	0.73	0.61	0.76	0.53	0.67	0.61
		H'	2.27	1.68	1.74	1.60	1.57	1.81	2.02	0.99	1.88	1.10	1.46	1.35
		D	0.86	0.69	0.77	0.65	0.70	0.72	0.81	0.55	0.79	0.60	0.67	0.64
DNA Barcoding		# of Larvae (N)	2	5	4	3	2	1	10	0	11	1	4	1
		# of Taxa (N)	2	5	2	3	2	1	7	0	10	1	4	1
Morphological Analysis		# of Larvae (N)	10	11	5	6	4	5	10	7	4	6	1	4
		# of Taxa (N)	5	5	4	5	3	3	5	5	3	4	1	3
		J	0.88	0.96	0.96	0.97	0.95	0.87	0.88	0.92	0.95	0.96	1.00	0.95
		H'	1.42	1.55	1.33	1.56	1.04	0.95	1.42	1.48	1.04	1.33	0.00	1.04
		D	0.72	0.78	0.72	0.78	0.63	0.56	0.72	0.73	0.63	0.72	0.00	0.63
