# Supplementary material for: Larval assemblages over the abyssal plain in the Pacific are highly diverse and spatially patchy
Source: PeerJ. 2019 Sep 26;7:e7691. doi: 10.7717/peerj.7691 (PMC6766376; doi:10.7717/peerj.7691)
Supplement: Table S2 — References are listed in File S2. [file peerj-07-7691-s002.pdf]

Region	Fwd. Primer ID	Fwd. Primer Sequence (5'-3')	Rev. Primer ID	Rev. Primer Sequence (5'-3')	Amplicon Length (bp)	Reference
**A)**						
18S_V1&2	F04	GCTTGTCTCAAAGATTAAGCC	R22	GCCTGCTGCCTTCCTTGGA	~365	90
18S_V7&8	3F	GYGGTGCATGGCCGTTSKTRGTT	5RC	GTGTGYACAAAGGBCAGGGAC	~320-330	91
mtCOI	mlCOIintF	GGWACWGGWTGAACWGTWTAYCCYCC	jgHCO2198	TAIACYTCIGGRTGICCRAARAAYCA	~313	92
**B)**						
mtCOI	LCO1490	GGTCAACAAATCATAAAGATATTGG	HCO2198	TAAACTTCAGGGTGACCAAAAAATCA	~650	93
mtCOI	polyLCO	GAYTATWTTCAACAAATCATAAAGATATTGG	polyHCO	TAMACTTCWGGGTGACCAAARAATCA	~650	94
18S FR1	18SA	AYCTGGTTGATCCTGCCAGT	1324R	CGGCCATGCACCACC	~1800	95–97
18S FR2	620F	TAAAGYTGYTGCAGTTAAA	18SB	ACCTTGTTACGACTTTTACTTCCTC		
