# Supplementary material for: Larval assemblages over the abyssal plain in the Pacific are highly diverse and spatially patchy
Source: PeerJ. 2019 Sep 26;7:e7691. doi: 10.7717/peerj.7691 (PMC6766376; doi:10.7717/peerj.7691)
Supplement: Table S3 [file peerj-07-7691-s003.pdf]

Type	Name	DOI/SRA accession #	Content
Metadata File	OTU_Table_AbundInfo_All.xlsx	doi:10.5061/dryad.vb68g9d	Overview (1 tab for each marker) of presence/absence of OTUs at sampling stations
Metadata File	OTU_Table_TaxInfo_All.xlsx	doi:10.5061/dryad.vb68g9d	Taxonomic identity of each OTU (1 tab for each marker)
Sequence File	mtCOI.OTUrepsequences.fasta	doi:10.5061/dryad.vb68g9d	Representative nucleotide sequence of each OTU captured with the mtCOI marker
Sequence File	mtCOI.unique.chimremov.fastq.gz	SRR9304915	Raw mtCOI sequencing data without duplicates and chimeras
Sequence File	18S_V1_2.OTUrepsequences.fasta	doi:10.5061/dryad.vb68g9d	Representative nucleotide sequence of each OTU captured with the 18S_V1&2 marker
Sequence File	18S_V1_2.unique.chimremov.fastq.gz	SRR9304914	Raw 18S_V1&2 sequencing data without duplicates and chimeras
Sequence File	18S_V7_8.OTUrepsequences.fasta	doi:10.5061/dryad.vb68g9d	Representative nucleotide sequence of each OTU captured with the 18S_V7&8 marker
Sequence File	18S_V7_8.unique.chimremov.fastq.gz	SRR9304913	Raw 18S_V7&8 sequencing data without duplicates and chimeras
