# Supplementary material for: Larval assemblages over the abyssal plain in the Pacific are highly diverse and spatially patchy
Source: PeerJ. 2019 Sep 26;7:e7691. doi: 10.7717/peerj.7691 (PMC6766376; doi:10.7717/peerj.7691)
Supplement: Table S4 — PERMANOVA with distance matrix, ANOSIM, MRPP, and Mantel tests were performed for each marker with 999 permutations for the presence/absence and non-transformed (in parentheses) datasets. Multivariate homogeneity of groups dispersions was tested with the betadisper and anova functions in R (999 permutations) to assess the validity of the ANOSIM tests. The indeces of species richness, evenness, and diversity are mean values and significance was determined with t-tests. S(obs) = No. of OTUs observed. H′= Shannon-Weaver index. D = Simpson Diversity index. J = Pielou’s evenness index. [file peerj-07-7691-s004.pdf]

Marker	PERMANOVA		ANOSIM		BetaDisper		MRPP		Mantel		***S(obs)***			***J'***			***H'***			***D***		
	R^2	*p-value*	*R*	*p-value*	*F*	*p-value*	*A*	*p-value*	*r*	*p-value*	*UK*	*OMS*	*p-value*	*UK*	*OMS*	*p-value*	*UK*	*OMS*	*p-value*	*UK*	*OMS*	*p-value*
18S_V1&2	0.099	0.362	0.048	0.325	0.054	0.814	0.003	0.446	-0.064	0.752	20.333	13.333	0.050	0.617	0.679	0.553	1.852	1.694	0.656	0.687	0.697	0.927
	(-0.103)	(0.149)	(-0.103)	(0.140)	(0.000)	(0.994)	(0.008)	(0.144)	(-0.116)	(0.835)												
18S_V7&8	0.097	0.420	0.094	0.235	0.133	0.727	0.002	0.460	-0.072	0.723	25.000	20.833	0.382	0.803	0.799	0.921	2.536	2.370	0.483	0.863	0.858	0.894
	(0.092)	(0.423)	(-0.023)	(0.575)	(0.358)	(0.636)	(0.001)	(0.410)	(-0.158)	(0.992)												
mtCOI	0.113	0.212	0.098	0.182	0.314	0.573	0.008	0.263	-0.072	0.767	13.333	9.833	0.108	0.693	0.651	0.424	1.776	1.467	0.151	0.729	0.678	0.345
	(0.092)	(0.387)	(0.026)	(0.308)	(0.116)	(0.739)	(0.001)	(0.378)	(-0.091)	(0.819)												
