# Supplementary material for: Larval assemblages over the abyssal plain in the Pacific are highly diverse and spatially patchy
Source: PeerJ. 2019 Sep 26;7:e7691. doi: 10.7717/peerj.7691 (PMC6766376; doi:10.7717/peerj.7691)
Supplement: Table S5 — Specimens were taxonomically classified via a consensus call using the SILVA database, NCBI Genbank and the Statistical Assignment Package. Metabarcoding OTUs from each marker were classified applying the same methods to representative sequences for each OTU. *No bivalve OTUs in the 18S_V1&2 metabarcoding dataset. ** a sipunculid. [file peerj-07-7691-s005.pdf]

ID	Barcoding			Nearest Metabarcoding OTU						Most likely Consensus		
	18S V1&2	18S V7&8	mtCOI	18S V1&2		18S V7&8		mtCOI		Family	Genus	Species/Classification
				Taxonomy	%ID	Taxonomy	%ID	Taxonomy	%ID			
**A) GASTROPODA**												
MG1	*Vetigastropoda sp.*	*Bathyxylophila sp.*	*Vetigastropoda sp.*	*Fluxinella sp.*	88.1	*Vetigastropoda sp.*	91.9	-	-	Larocheidae	Bathyxylophila	*Bathyxylophila sp.*
MG2	*Vetigastropoda sp.*	*Calliotropidae sp.*	*Gastropoda sp.*	*Vetigastropoda sp.*	97.5	*Calliotropidae sp.*	98.5	-	-	Calliotropidae	-	*Calliotropidae sp.*
MG4	*Vetigastropoda sp.*	*Vetigastropoda sp.*	*Gastropoda sp.*	*Vetigastropoda sp.*	97.5	*Vetigastropoda sp.*	99.1	-	-	-	-	*Vetigastropoda sp.*
MG5	*Fluxinella sp.*	*Vetigastropoda sp.*	-	*Vetigastropoda sp.*	100	*Vetigastropoda sp.*	100	-	-	Seguenziidae	Fluxinella	*Fluxinella sp.*
MG6	*Eukaryota sp.*	-	-	*Vetigastropoda sp.*	76.8	-	-	-	-	-	-	*Eukaryota sp.*
MG8	*Fluxinella sp.*	*Vetigastropoda sp.*	*Gastropoda sp.*	*Vetigastropoda sp.*	99.2	*Vetigastropoda sp.*	97.9	-	-	Seguenziidae	Fluxinella	*Fluxinella sp.*
MG10	*Vetigastropoda sp.*	*Bathyxylophila sp.*	-	*Vetigastropoda sp.*	94.8	*Vetigastropoda sp.*	96.4	-	-	Larocheidae	Bathyxylophila	*Bathyxylophila sp.*
MG11	*Vetigastropoda sp.*	-	-	*Vetigastropoda sp.*	95.8	-	-	-	-	-	-	*Vetigastropoda sp.*
MG12	*Vetigastropoda sp.*	*Calliotropidae sp.*	-	*Vetigastropoda sp.*	98.9	*Calliotropidae sp.*	99.1	-	-	Calliotropidae	-	*Calliotropidae sp.*
MG13	*Vetigastropoda sp.*	*Calliotropidae sp.*	*Holozoa sp.*	*Vetigastropoda sp.*	95.6	*Calliotropidae sp.*	97.6	-	-	Calliotropidae	-	*Calliotropidae sp.*
MG14	*Fluxinella sp.*	*Vetigastropoda sp.*	-	*Fluxinella sp.*	98.6	*Vetigastropoda sp.*	98.8	-	-	Seguenziidae	Fluxinella	*Fluxinella sp.*
MG15	*Fluxinella sp.*	*Vetigastropoda sp.*	*Holozoa sp.*	*Vetigastropoda sp.*	96.7	*Calliotropidae sp.*	93.2	-	-	Seguenziidae	Fluxinella	*Fluxinella sp.*
MG16	*Ventsia tricarinata*	*Ventsia tricarinata*	*Holozoa sp.*	*Vetigastropoda sp.*	95.9	*Vetigastropoda sp.*	95.5	-	-	Seguenziidae	Ventsia	*Ventsia tricarinata*
MG17	*Vetigastropoda sp.*	*Calliostoma sp.*	-	*Vetigastropoda sp.*	99.2	*Calliotropidae sp.*	98.8	-	-	Calliostomatidae	Calliostoma	*Calliostoma sp.*
**B) BIVALVIA**												
MB1	*Propeamussiidae sp.*	*Propeamussium sp.*	-	N.A.*	N.A.*	*Nuculanida sp.*	89.1	-	-	Propeamussiidae	Propeamussium	*Propeamussium sp.*
MB3	*Yoldiella sp.*	*Yoldiella sp.*	-	N.A.*	N.A.*	*Nuculanida sp.*	90.2	-	-	Yoldiidae	Yoldiella	*Yoldiella sp.*
MB5	*Teredinidae sp.*	*Xylophagidae sp.*	*Holozoa sp.*	N.A.*	N.A.*	*Nuculanida sp.*	83.3	-	-	Xylophagidae	-	*Xylophagidae sp.*
**C) POLYCHAETA and SIPUNCULA**												
FR6	*Dysponetus caecus*	*Dysponetus caecus*	-	*Aurospio dibranchiata*	94.7	*Aurospio dibranchiata*	94.1	-	-	Chrysopetalidae	Dysponetus	*Dysponetus caecus*
FR7	*Dysponetus caecus*	*Dysponetus caecus*	-	*Aurospio dibranchiata*	94.7	*Aurospio dibranchiata*	94.1	-	-	Chrysopetalidae	Dysponetus	*Dysponetus caecus*
PA1	*Laonice sp.*	*Laonice sp.*	*Laonice sp.*	*Aonides selvagensis*	85.6	*Aurospio dibranchiata*	91.0	-	-	Spionidae	Laonice	*Laonice sp.*
PA2	*Sirsoe sirikos*	*Sirsoe sirikos*	*Phyllodocida sp.*	*Phyllochaetopterus limicolus*	91.2	*Nerillidae sp.*	95.0	-	-	Hesionidae	Sirsoe	*Sirsoe sirikos*
PA4	*Austropolaria magnicirrata*	*Austropolaria magnicirrata*	*Polychaeta sp.*	*Polynoidae sp.*	99.2	*Phyllodocida sp.*	93.1	-	-	Polynoidae	Austropolaria	*Austropolaria magnicirrata*
PA7	*Austropolaria magnicirrata*	*Austropolaria magnicirrata*	*Polychaeta sp.*	*Polynoidae sp.*	99.2	*Phyllodocida sp.*	93.1	-	-	Polynoidae	Austropolaria	*Austropolaria magnicirrata*
PA10	*Fauveliopsis sp.*	*Fauvelopsis scabra*	-	*Aonides selvagensis*	91.2	*Nerillidae sp.*	94.0	-	-	Fauveliopsidae	Fauveliopsis	*Fauveliopsis scabra*
SI1	*Capitella sp.*	*Phascolosoma sp.***	-	*Capitella sp.*	100	*Capitella sp.*	89.6	-	-	Phascolosomatidae	Phascolosoma	*Phascolosoma sp.***
PL2	*Spionida sp.*	*Spionida sp.*	*Polychaeta sp.*	*Aurospio dibranchiata*	88.7	*Aurospio dibranchiata*	96.6	-	-	-	-	*Spionida sp.*
PL3	*Sirsoe sirikos*	*Sirsoe sirikos*	*Holozoa sp.*	*Phyllochaetopterus limicolus*	91.5	*Nerillidae sp.*	95.6	-	-	Hesionidae	Sirsoe	*Sirsoe sirikos*
PL4	*Spionida sp.*	*Spionidae sp.*	*Polychaeta sp.*	*Aurospio dibranchiata*	91.2	*Aurospio dibranchiata*	96.6	-	-	Spionidae	-	*Spionidae sp.*
PL5	*Spionida sp.*	*Spionidae sp.*	-	*Aurospio dibranchiata*	91.2	*Aurospio dibranchiata*	96.5	-	-	Spionidae	-	*Spionidae sp.*
PL7	*Spionida sp.*	*Spionidae sp.*	-	*Aurospio dibranchiata*	91.2	*Aurospio dibranchiata*	96.6	-	-	Spionidae	-	*Spionidae sp.*
PL8	*Spionida sp.*	*Spionidae sp.*	-	*Aurospio dibranchiata*	91.2	*Aurospio dibranchiata*	96.6	-	-	Spionidae	-	*Spionidae sp.*
PL10	*Macellicephala gloveri*	*Macellicephala gloveri*	-	*Polynoidae sp.*	97.1	*Spionida sp.*	89.4	-	-	Polynoidae	Macellicephala	*Macellicephala gloveri*
PL11	*Spionida sp.*	*Spionidae sp.*	-	*Aurospio dibranchiata*	91.2	*Aurospio dibranchiata*	96.6	-	-	Spionidae	-	*Spionidae sp.*
PL12	*Spionida sp.*	-	-	*Aurospio dibranchiata*	91.2	-	-	-	-	-	-	*Spionida sp.*
PL14	*Dysponetus caecus*	*Dysponetus caecus*	-	*Aurospio dibranchiata*	94.7	*Aurospio dibranchiata*	94.1	-	-	Chrysopetalidae	Dysponetus	*Dysponetus caecus*
SI15	*Capitella sp.*	*Phascolosoma sp.***	*Capitella sp.*	*Capitella sp.*	100	*Capitella sp.*	89.6	*Capitella sp.*	100	Phascolosomatidae	Phascolosoma	*Phascolosoma sp.***
PL16	*Glandulospio orestes*	*Spionida sp.*	-	*Aurospio dibranchiata*	87.6	*Aurospio dibranchiata*	91.3	-	-	Spionidae	Glandulospio	*Glandulospio orestes*
PL18	*Spionida sp.*	*Spionidae sp.*	-	*Aurospio dibranchiata*	91.2	*Aurospio dibranchiata*	96.6	-	-	Spionidae	-	*Spionidae sp.*
PL20	*Spionida sp.*	*Spionidae sp.*	-	*Aurospio dibranchiata*	91.2	*Aurospio dibranchiata*	96.6	-	-	Spionidae	-	*Spionidae sp.*
PL22	*Spionida sp.*	*Spionidae sp.*	-	*Aurospio dibranchiata*	91.2	*Aurospio dibranchiata*	96.6	-	-	Spionidae	-	*Spionidae sp.*
PL23	*Neogyptis julii*	*Phyllodocida sp.*	-	*Phyllochaetopterus limicolus*	92.6	*Nerillidae sp.*	93.1	-	-	Hesionidae	Neogyptis	*Neogyptis julii*
PL33	*Dysponetus caecus*	*Dysponetus caecus*	-	*Aurospio dibranchiata*	93.9	*Aurospio dibranchiata*	94.1	*Sabellida sp.*	83.333	Chrysopetalidae	Dysponetus	*Dysponetus caecus*
