# Supplementary material for: Larval assemblages over the abyssal plain in the Pacific are highly diverse and spatially patchy
Source: PeerJ. 2019 Sep 26;7:e7691. doi: 10.7717/peerj.7691 (PMC6766376; doi:10.7717/peerj.7691)
Supplement: Table S6 — Information on the ecology or existing sample records are included for taxa classified to the family level and beyond. References are listed in File S2. [file peerj-07-7691-s006.pdf]

Phylum	Subphylum	Class	Order	Family	OTU ID / Species	Ecology, Habitat or Records in the Literature	Ref(s).
Mollusca	-	Gastropoda	Vetigastropoda	-	*Vetigastropoda sp. A-F*	-	-
Mollusca	-	Gastropoda	Vetigastropoda	-	*Trochoidea sp.*	-	-
Mollusca	-	Bivalvia	-	-	*Bivalvia sp. A-B*	-	-
Annelida	-	Polychaeta	-	-	*Chaetosphaerid sp.*	-	-
Annelida	-	Polychaeta	Terebellida	Fauveliopsidae	*Fauveliopsidae sp.*	Family has been recorded from abyssal depths in the Pacific Ocean.	22,86
Annelida	-	Polychaeta	Phyllodocida	Sigalionidae	*Sigalionidae sp.*	Family has been found on the abyssal plain in the CCZ.	9,53
Annelida	-	Polychaeta	Phyllodocida	Polynoidae	*Polynoidae sp.*	Family has been recorded from the abyssal plain in the CCZ.	27
Annelida	-	Polychaeta	-	-	*Polychaeta sp. A-B*	-	-
Echinodermata	Asterozoa	Ophiuroidea	-	-	*Ophiuroidea sp. A*	-	-
Bryozoa	Gymnolaemata	-	Ctenostomatida	-	*Ctenostomatida sp.*	-	-
Arthropoda	Crustacea	Copepoda	Cyclopoida	Schminkepinellidae	*Barathricola sp. 1-2*	Recorded from deep-sea hydrothermal vent sites and the hyperbenthos.	11,87
Arthropoda	Crustacea	Copepoda	Harpacticoida	Aegisthidae	*Pontostratiotes sp. 1-2*	Recorded in the abyssal North East Atlantic and described from hydrothermal vents and benthic trawls.	2,3
Arthropoda	Crustacea	Copepoda	Harpacticoida	Argestidae	*Argestidae sp.*	Observed at or near woodfalls at abyssal depths in the CCZ.	24
Arthropoda	Crustacea	Copepoda	Harpacticoida	Zosimeidae	*Zosimeidae sp.*	Recorded from abyssal depths in the Western Atlantic and North West Pacific.	88
Arthropoda	Crustacea	Copepoda	Harpacticoida	Ectinosomatidae	*Ectinosomatidae sp.*	Only *Bradya* is represented in the deep sea (e.g. the abyssal plains in the Angola Basin).	2,39
Arthropoda	Crustacea	Copepoda	Harpacticoida	Cerviniidae	*Cerviniidae sp.*	Observed at or near woodfalls at abyssal depths in the CCZ.	24
Arthropoda	Crustacea	Copepoda	Harpacticoida	Pseudotachidiidae	*Pseudotachidiidae sp.*	Observed at or near woodfalls at abyssal depths in the CCZ.	24
Arthropoda	Crustacea	Copepoda	Harpacticoida	Ameiridae	*Ameiridae sp.*	Occurs at depths down to 6300m. Some members are loose symbionts on invertebrates.	2,12
Arthropoda	Crustacea	Peracarida	Amphipoda	Uristidae	*Abyssorchomene sp.*	Abyssal necrophagous amphipod, previously reported from the Atlantic and Pacific.	49,50
Arthropoda	Crustacea	Peracarida	Amphipoda	Cyclocaridae	*Cyclocaris sp.*	Scavenger genus found in baited traps at abyssal depths including the North Pacific.	89
Arthropoda	Crustacea	Tantulocarida	-	-	*Tantulocarida sp.*	-	-
