# Supplementary figures and images for: Larval assemblages over the abyssal plain in the Pacific are highly diverse and spatially patchy
Source: PeerJ. 2019 Sep 26;7:e7691. doi: 10.7717/peerj.7691 (PMC6766376; doi:10.7717/peerj.7691)

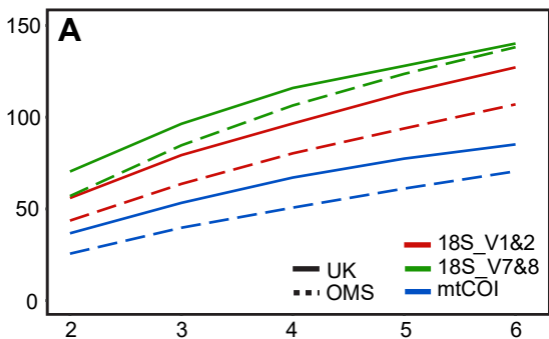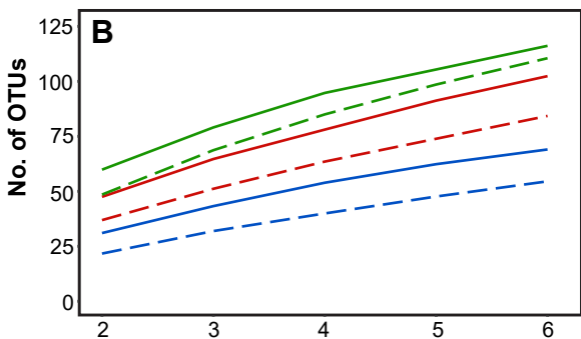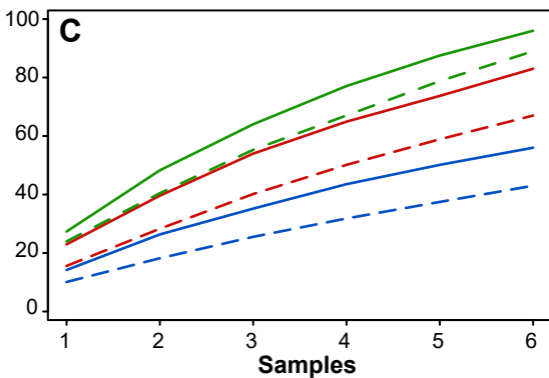

Supplement: Figure S1 — (A) Jackknife 1, (B) Bootstrap and (C) Species accumulation curves compared between markers and sampling strata. All plots include both barcoding and metabarcoding data. [file peerj-07-7691-s007.pdf]

**A****18S\_V1&2**  
(34)**18S\_V7&8**  
(41)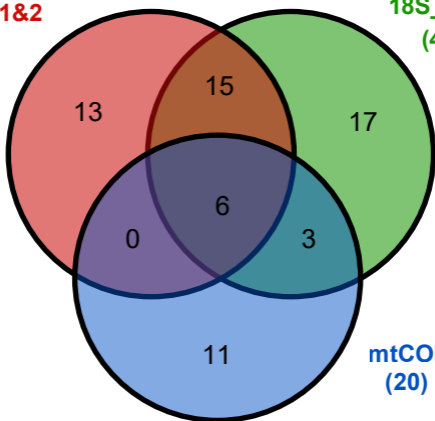**B****18S\_V1&2**  
(29)**18S\_V7&8**  
(29)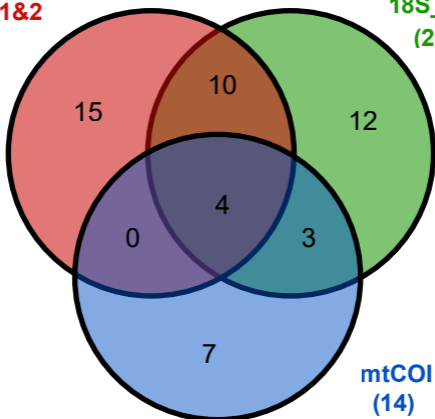

Supplement: Figure S3 — Numbers in parentheses represent the total number of meroplankton families (A) and genera (B) captured by each marker. The number of families is larger than the number of genera, due to many OTUs being classified only to family. [file peerj-07-7691-s009.pdf]

# 18S\_V7&8

# mtCOI

**A**

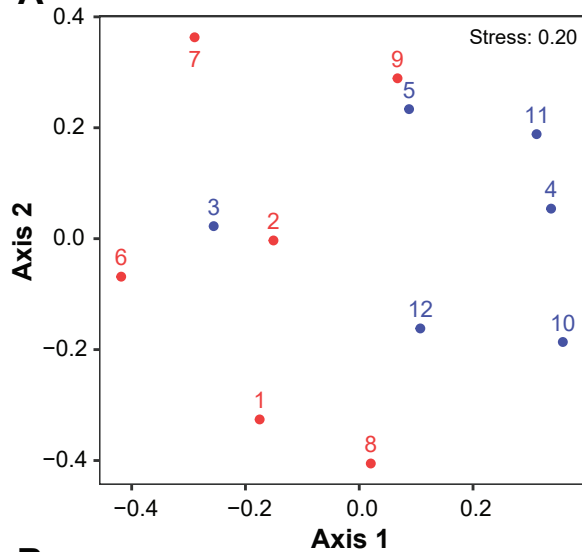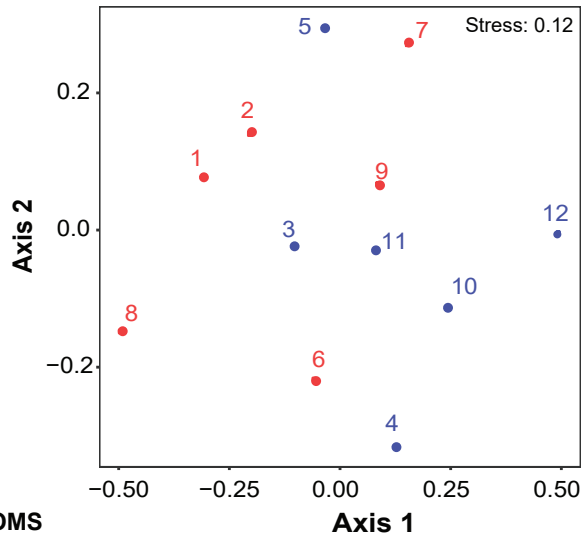

**B**

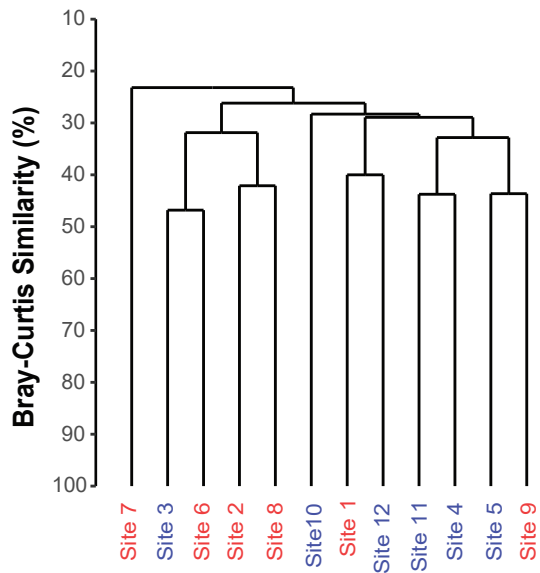

OMS

UK

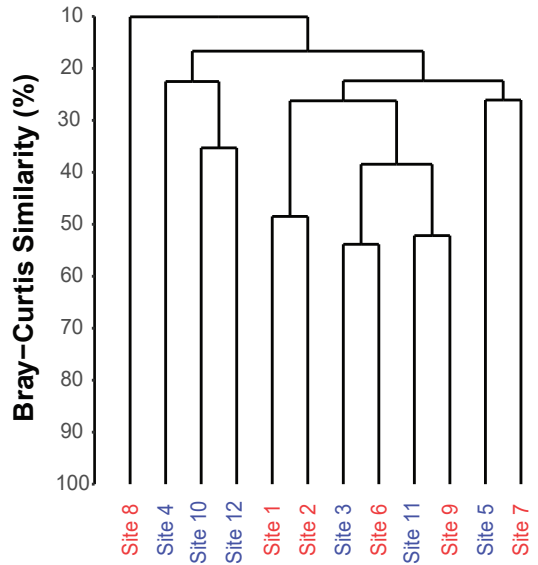

Supplement: Figure S4 — The non-parametric multi-dimensional scaling (NMDS) plot (A) and cluster dendrogram (B) were constructed for the 18S_V7&8 and mtCOI marker. The NMDS plot show the placement of 12 abyssal sites (UK1 + OMS1) in ordination space, colored by strata. The dendrogram is based on a hierarchical cluster analysis using group average linkage and the Bray-Curtis distance measure. OTU sequence abundance was transformed into presence/absence prior to both analyses. [file peerj-07-7691-s010.pdf]

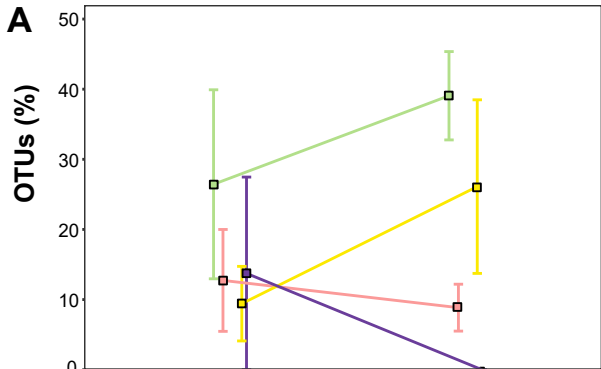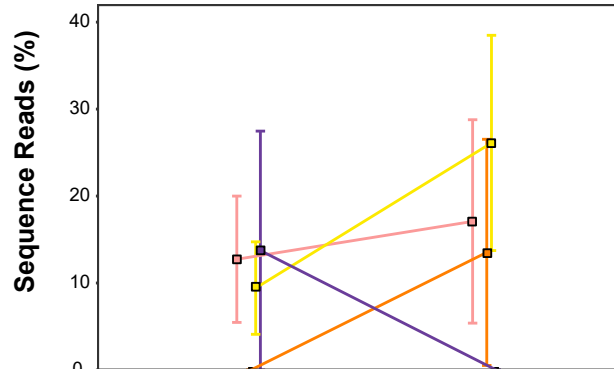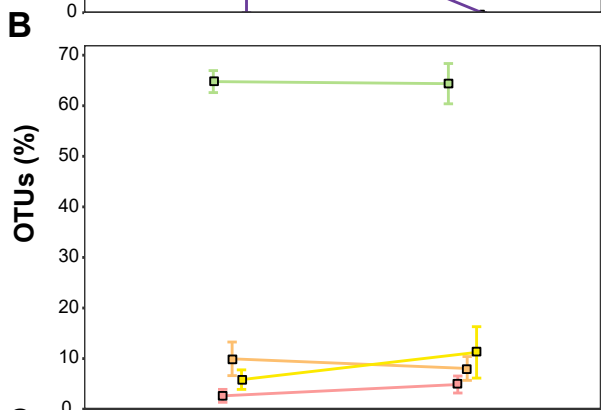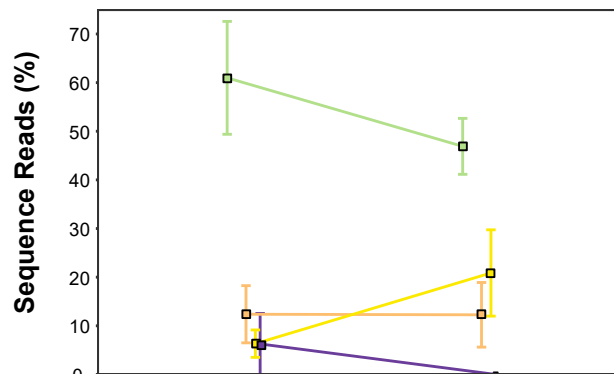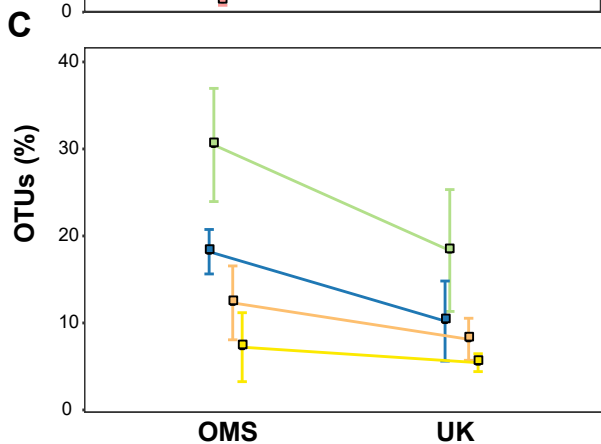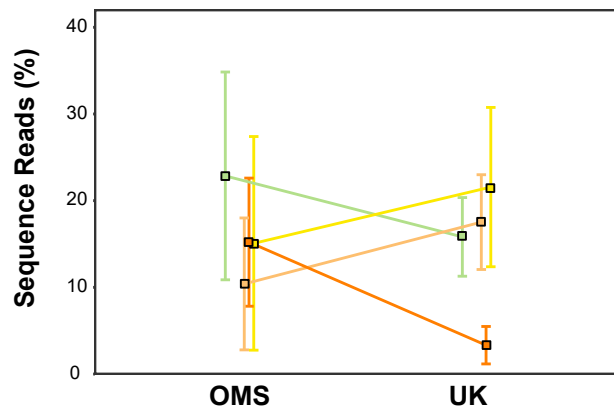

— Bryozoa — Copepoda — Gastropoda — Peracarida — Polychaeta — Rhabditophora — Others

Supplement: Figure S5 — Proportion of operational taxonomic units (% OTUs) and % sequence reads are shown for the four most abundant taxa captured by (A) 18S_V1&2, (B) 18S_V7&8, and (C) mtCOI. Error bars show standard error. Nematoda (0.7% of all meroplankton OTUs combining the three metabarcoding datasets), Tantulocarida (0.7%), Tunicata (2.2%), Vertebrata (3.6%), Chitonida (0.7%), Xenacoelomorpha (0.4%), Decapoda (0.4%), Pedunculata (1.1%), Pycnogonida (0.4%), Entoprocta (1.4%), Nemertea (0.7%), Scaphopoda (0.4%), and Sipuncula (0.4%) were grouped into Others (12.9% total). [file peerj-07-7691-s011.pdf]
