# Supplementary material for: Larval assemblages over the abyssal plain in the Pacific are highly diverse and spatially patchy
Source: PeerJ. 2019 Sep 26;7:e7691. doi: 10.7717/peerj.7691 (PMC6766376; doi:10.7717/peerj.7691)
Supplement: Figure S2 — (A) proportion of OTUs (%), and (B) % sequence reads are shown for 10 dominant taxonomic groups with meroplanktonic representatives. Nematoda (0.7% of all meroplankton OTUs combining the three metabarcoding datasets), Tantulocarida (0.7%), Tunicata (2.2%), Vertebrata (3.6%), Chitonida (0.7%), Xenacoelomorpha (0.4%), Decapoda (0.4%), Pedunculata (1.1%), Pycnogonida (0.4%), Entoprocta (1.4%), Nemertea (0.7%), Scaphopoda (0.4%), and Sipuncula (0.4%) were grouped into Others (12.9% total). Labels for 18S markers V1&2 and V7&8 are abbreviated. [file peerj-07-7691-s008.pdf]

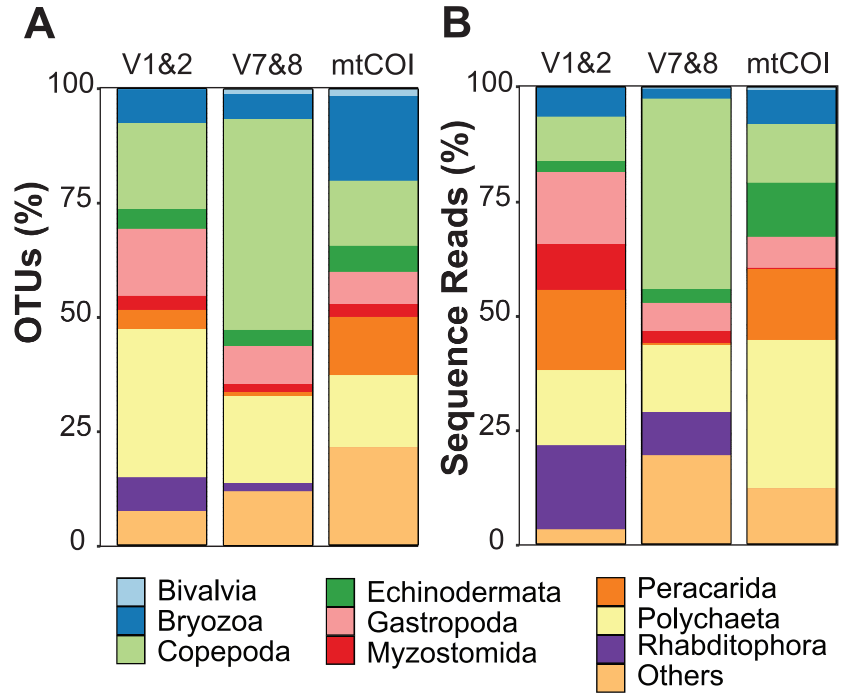

A
B
V1&2
V7&8
mtCOI
OTUs (%)
Sequence Reads (%)
100
75
50
25
0
Bivalvia
Bryozoa
Copepoda
Echinodermata
Gastropoda
Myzostomida
Peracarida
Polychaeta
Rhabditophora
Others
