# Supplementary material for: Larval assemblages over the abyssal plain in the Pacific are highly diverse and spatially patchy
Source: PeerJ. 2019 Sep 26;7:e7691. doi: 10.7717/peerj.7691 (PMC6766376; doi:10.7717/peerj.7691)
Supplement: File S1 [file peerj-07-7691-s012.docx]

**Supplementary File 1 for:**

**Larval assemblages over the abyssal plain in the Pacific are highly diverse and spatially patchy**

Oliver Kersten, Eric W. Vetter, Michelle J. Jungbluth, Craig R. Smith, Erica Goetze

**Supplemental Material and Methods:**

*Sample Collection:*

Study sites were sampled with two plankton pumps (McLane Large Volume Water Transfer System WTS-LV30; McLane Research Laboratories) mounted on a free-vehicle (above the structure of the free vehicle to avoid flow blockage) to autonomously sample zooplankton at ~3 m above the seafloor. Pumping occurred at a rate of ~ 27 L min^-1^ through a 3-cm inlet located near the top of the pump, yielding a total sample volume of on average 33.66 m^3^ of seawater filtered per pump and deployment (Table S1).

Epipelagic zooplankton samples were collected at three stations in the UK01 stratum and one station in the OMS01 stratum by towing a 1 m^2^ net obliquely (202 μm mesh size) during both night (two tows) and day (two tows) from a maximum depth of approximately 200 - 300 m to the surface. This sampling was conducted in order to assess any unintentional zooplankton capture during ascent and descent of the plankton pump lander. On average, a volume of 610.5 m^3^ of seawater was sampled during each tow, which lasted ~ 30 minutes. Day tows were conducted at 1 pm and night tows were performed between 1 am – 6 am. Upon recovery, subsequent handling of the samples, including quantitative splitting and preservation, was conducted as in the plankton pump samples.

*Metabarcoding:*

Prior to DNA extraction, the four epipelagic samples were size-fractionated into four size classes, 0.2 – 0.5 mm, 0.5 – 1 mm, 1 - 2 mm, and > 2 mm. DNA was bulk extracted from each sample using the E.Z.N.A. HP Tissue DNA Maxi kit (OMEGA), following the manufacturer’s protocol. For epipelagic samples, DNA was eluted 3X and 500 μl of the first elution of each size fraction was combined to make 2 ml of each sample.

Two regions of the nuclear 18S rRNA gene were amplified by PCR using the primer combinations (1) F04 and R22 (~365 bp fragment)(Fonseca et al. 2010), and (2) 3F and 5RC (~325 bp fragment)(Machida and Knowlton 2012). Resulting amplicons spanned the variable regions V1&V2 (Fonseca et al. 2010), and V7&V8 (Machida and Knowlton 2012). A ~313 bp fragment of the mitochondrial cytochrome *c* oxidase subunit I (mtCOI) gene was amplified using the primer combination mlCOIintF and jgHCO2198 (Leray et al. 2013) (Table S2). PCR amplification was performed in triplicate 20 μl reactions, each containing 1 μl of genomic DNA, 10 μl of MangoMix (Bioline), 1 μl of Bovine Serum Albumin (10 mg/ml, BSA, New England Biolabs), and 0.6 μl (mtCOI) - 1.2 μl (18S) each of forward and reverse primer (10 μM). The PCR conditions consisted of 2 min. denaturation at 95°C followed by 40 cycles of 30-45 s at 95°C (18S: 45 s, mtCOI: 30 s), 30-45 s at 50-55°C (18S: 45s at 55°C, mtCOI: 30 s at 50°C), 1 min at 72°C, with a final extension step of 5-10 min (18S: 10 min, mtCOI: 5min) at 72°C. Aerosol barrier pipette tips were used and negative controls were included for all amplifications. PCR products were pooled for each sample and marker to reduce stochastic differences in amplification across reactions, and purified using the Qiagen MinElute kit, with elution in 10µl of dH2O. DNA concentrations were quantified using the Qubit dsDNA Broad-Range Assay Kit (Life Technologies), normalized across markers, and all PCR products from each of the 18 samples (four epipelagic, 14 abyssal – two pumps at first two sites) were pooled into a single DNA template for library preparation.

Prior to amplicon analysis, custom reference databases for the alignment and taxonomic inferences of mtCOI sequences in downstream analyses was built. The MIDORI_Longest database (Machida et al. 2017) was downloaded and dereplicated. Contrary to e.g. OTU clustering steps, kmer-based taxonomic inference does not require an aligned reference database and, in order to maintain taxonomic coverage across as many taxa possible, the unaligned MIDORI_Longest database was used, containing 110,619 unique sequences across 30 phyla. For downstream analyses steps requiring an aligned custom reference database, the dereplicated sequences were translated to amino acids (using the appropriate translation code) using Multiple Alignment of Coding Sequences (MACSE)(Ranwez et al. 2011)(-prog translateNT2AA). All Insecta sequences, as well as sequences containing stop codons, were discarded. The remaining sequences were aligned using MUSCLE (Edgar 2004), and the alignment was visually assessed and manually improved using Geneious R11 (Kearse et al. 2012) and JalView2 (Waterhouse et al. 2009) prior to another refinement step in MUSCLE (-refine). The final alignment was then back-translated to nucleotides using MACSE (-prog reportGapsAA2NT), for compatibility in downstream analyses. The final aligned reference database contained 23,617 sequences covering a total of 27 phyla.

Reads from all three markers were trimmed and filtered for sequence quality above a PHRED score of 20 using cutadapt (Martin 2011). Sequences were demultiplexed based on primer sequences using the program Sabre (https://github.com/najoshi/sabre), and R1 and R2 reads were checked for appropriate pairing using FastqPairedEndValidator (http://www.mcdonaldlab.biology.gatech.edu/seqtools_frame.htm). Paired reads were merged in PEAR (v 0.9.6, Zhang et al. 2014), filtering out sequences < 250 bp in length and requiring a minimum overlap of 100 bp between forward and reverse reads. Merged sequences containing the reverse primer on the 5’ end were reverse-complemented to obtain all sequence reads in the same orientation for downstream analyses. Subsequent steps were performed in mothur (v1.38.0), with guidelines as outlined in the MiSeq SOP (Schloss et al. 2009; Kozich et al. 2013). Within mothur, primers were trimmed (trim.seqs with oligo flag) and sequences were filtered with the following parameters: no ambiguous bases, maximum homopolymer length of 10 bp, and sequence length between 295-355 bp (18S_V7&8), 335-395 bp (18S_V1&2), and 279-339 bp (mtCOI). Unique 18S sequences were filtered and aligned to the SILVA128 database (Quast et al. 2013; Yilmaz et al. 2014)(search=kmer, ksize=8, align=needleman, match=+1, mismatch=-1, gapopen=-2, gapextend=-1, threshold=0.5, flip=F), and trimmed to remove sequences that aligned outside the target region. For the mtCOI amplicon, sequences were aligned to the aligned custom reference database using the option “enrichAlignment” in MACSE, and trimmed to remove sequences that aligned outside the target region. Duplicate sequences of all three markers were removed again, and unique sequences were pre-clustered at 99% (18S) and 97% (mtCOI) similarity (diffs=2, diffs=1 & diffs=4; match=1, mismatch=-1, gapopen=-2, gapextend=-1), resulting in 471,100 (18S_V1&2), 390,979 (18S_V7&8) and 35,837 (mtCOI) remaining sequences. Chimeras were identified and removed using VSEARCH (Rognes et al. 2016), as implemented in mothur. Non-chimeric sequences were assigned taxonomy based on the eukaryotic portion of the Silva128 database (18S_V1&2 region - 33,864 eukaryotic sequences, 18S_V7&8 region - 42,065 eukaryotic sequences), as well as the unaligned custom reference database (mtCOI), using a naïve Bayesian classifier (Wang method) with taxonomic levels at < 80% bootstrap support discarded (Wang et al. 2007) (parameters: method=wang, ksize=8, iters=100, cutoff=80). Sequences assigned taxonomy within Holozoa were retained for further analyses. The remaining sequences (18S_V1&2 – 229,056, 18S_V7&8 – 128,732, mtCOI – 27,332) were clustered into OTUs (cluster.split, splitmethod=classify, taxlevel = 9) at 99% (18S) and 97% (mtCOI) similarity using the average neighbor method, and consensus taxonomy for each OTU was assigned. OTUs that were present in both abyssal and epipelagic samples were discarded, in order to remove sequences and OTUs that derive from overlying ecosystems (allochthonous to the deep sea), with OTUs present only in abyssal samples retained for further analyses (18S_V1&2 – 75,826 OTUs, 18S_V7&8 – 21,865 OTUs, mtCOI – 5,182 OTUs). In order to avoid inclusion of spurious low read count OTUs, OTUs with a relative abundance < 0.01% across all samples were removed for each marker. All stations were randomly subsampled (100X) to the lowest number of reads per station for each marker (3,681 - 18S_V1&2, 1229 – 18S_V7&8, 1288 – mtCOI) using Mothur in order to correct for sequencing bias (Kozich et al. 2013). This resulted in a total number of 44,089, 14,529 and 15,229 sequences and 794, 1219, and 1288 OTUs remaining for the 18S_V1&2, 18S_V7&8 and mtCOI markers, respectively. The median read count for each OTU at each station was used for further community analyses. The most common sequence within each OTU is hereinafter referred to as representative sequence.

For 18S OTUs, both the Silva taxonomy and blastn sequence similarity of the representative sequence were used to classify OTUs. Species names were reported for sequences with ≥ 99% sequence identity to the top hit in NCBI. In the absence of ≥ 99% sequence identity to any sequences in NCBI, ≥95% and ≥ 90% sequence similarity cutoffs, as well as the SILVA taxonomy, were used to assign OTUs to family or order level. For mtCOI OTUs, we used the amino acid structure of the marker, and additionally performed BLASTx searches of OTU representative sequences against the NCBI nt database. For mtCOI, a species name was assigned to an OTU if the top hit was within a) 97% (blastn) or b) 99% (blastx) sequence similarity. If these cutoffs were not reached, ≥ 90% and ≥ 85% sequence similarity to the top BLASTn hit, as well as the SILVA taxonomy, were used to assign OTUs to family or order level, respectively.

The majority of OTUs of the three markers could be categorized as holoplankton or meroplankton at the level of Class and Order; polychaete and mollusc classifications at family level were used to place these taxa by habitat. In the absence of a clear differentiation between holo- and meroplankton due to a lack of taxonomic resolution, a phylogenetic approach implemented in the Statistical Assignment Package (SAP) (Munch et al. 2008) was used to assign OTUs to finer taxonomic levels. Settings allowed for the inclusion of up to 40 homologs from GenBank with ≥70% sequence identity, and taxonomic assignments ≥ 80% posterior probability cutoff were accepted (Leray and Knowlton 2015). Taxonomic inferences for each OTU therefore include consideration of SILVA classification, the % identity to NCBI reference sequences and the SAP assignment (where applied).

*Larval DNA Barcoding:*

Subsamples (1/2 split) that were not used for the community analysis via microscopy presented in Kersten et al. (2017) were used to retrieve specimens for individual DNA barcoding prior to analyzing the remaining bulk sample via metabarcoding. Sorted specimens of larval polychaetes, gastropods, and bivalves were imaged using an Olympus model SZX16 microscope (8 – 110x magnification) and a Luminera Infinity-3 camera, to create a permanent morphological record of each specimen.

Genomic DNA was extracted using the DNeasy Blood & Tissue Kit (Qiagen), following manufacturer’s protocols with an overnight lysis, and DNA was eluted into 50 µl of AE buffer. Extract concentrations were measured on a Qubit fluorometer 2.0 (HS Assay), and all DNA extracts had measurable DNA (range 0.02 – 23.4 ng/ µl). Subsequently, fragments of 18S (~1800bp) and mtCOI (~650bp) were amplified by PCR. Primers used in PCR amplifications are reported in Table S2. These loci were chosen to facilitate comparison to concurrent taxonomic work on adults (e.g., Wiklund et al. 2017), as well as metabarcoding results described in this study. Amplifications were performed in 25 µl reaction volumes, with 0.75 µl of each primer [10 µM], 2 µl Bovine Serum Albumin (BSA, 1 mg/ml), 12.5 µl MangoMix (Bioline) and 1-2 µl of DNA extract. Cycling conditions were as follows: 95 °C for 5 min, followed by 35-40 cycles of 94 °C for 45 s, 45 – 57 °C for 45 s (45°C, 50°C mtCOI; 55°C, 57°C 18S rRNA), and 72 °C for 2 min, with a 10 min final extension at 72 °C. Successful amplifications were cleaned for sequencing using an Exosap protocol (Exonuclease - Shrimp Alkaline Phosphatase), and sequenced on a ABI3730XL capillary-based sequencer.

*Literature Cited:*

Edgar, R.C. 2004. MUSCLE: multiple sequence alignment with high accuracy and high throughput. *Nucleic Acids Research* **32** (5): 1792–1797.

Fonseca, V.G., Carvalho, G.R., Sung, W., Johnson, H.F., Power, D.M., Neill, S.P., Packer, M., Blaxter, M.L., Lambshead, P.J.D., and Thomas, W.K. 2010. Second-generation environmental sequencing unmasks marine metazoan biodiversity. *Nature Communications* **1**: 98.

Kearse, M., Moir, R., Wilson, A., Stones-Havas, S., Cheung, M., Sturrock, S., Buxton, S., Cooper, A., Markowitz, S., Duran, C., Thierer, T., Ashton, B., Meintjes, P., and Drummond, A. 2012. Geneious Basic: an integrated and extendable desktop software platform for the organization and analysis of sequence data. *Bioinformatics*  **28** (12): 1647–1649.

Kersten, O., Smith, C.R., and Vetter, E.W. 2017. Abyssal near-bottom dispersal stages of benthic invertebrates in the Clarion-Clipperton polymetallic nodule province. *Deep Sea Research Part I: Oceanographic Research Papers* **127**: 31–40.

Kozich, J.J., Westcott, S.L., Baxter, N.T., Highlander, S.K., and Schloss, P.D. 2013. Development of a dual-index sequencing strategy and curation pipeline for analyzing amplicon sequence data on the MiSeq Illumina sequencing platform. *Applied and Environmental Microbiology* **79** (17): 5112–5120.

Leray, M., and Knowlton, N. 2015. DNA barcoding and metabarcoding of standardized samples reveal patterns of marine benthic diversity. *Proceedings of the National Academy of Sciences of the United States of America* **112** (7): 2076–2081.

Leray, M., Yang, J.Y., Meyer, C.P., Mills, S.C., Agudelo, N., Ranwez, V., Boehm, J.T., and Machida, R.J. 2013. A new versatile primer set targeting a short fragment of the mitochondrial COI region for metabarcoding metazoan diversity: application for characterizing coral reef fish gut contents. *Frontiers in Zoology* **10**: 34.

Machida, R.J., and Knowlton, N. 2012. PCR primers for metazoan nuclear 18S and 28S ribosomal DNA sequences. *PloS One* **7** (9): e46180.

Machida, R.J., Leray, M., Ho, S.-L., and Knowlton, N. 2017. Metazoan mitochondrial gene sequence reference datasets for taxonomic assignment of environmental samples. *Scientific Data* **4**: 170027.

Martin, M. 2011. Cutadapt removes adapter sequences from high-throughput sequencing reads. *EMBnet.journal* **17** (1): 10–12.

Munch, K., Boomsma, W., Huelsenbeck, J.P., Willerslev, E., and Nielsen, R. 2008. Statistical assignment of DNA sequences using Bayesian phylogenetics. *Systematic Biology* **57** (5): 750–757.

Ranwez, V., Harispe, S., Delsuc, F., and Douzery, E.J.P. 2011. MACSE: Multiple Alignment of Coding SEquences accounting for frameshifts and stop codons. *PloS One* **6** (9): e22594.

Rognes, T., Flouri, T., Nichols, B., Quince, C., and Mahé, F. 2016. VSEARCH: a versatile open source tool for metagenomics. *PeerJ* **4**: e2584.

Schloss, P.D., Westcott, S.L., Ryabin, T., Hall, J.R., Hartmann, M., Hollister, E.B., Lesniewski, R.A., Oakley, B.B., Parks, D.H., and Robinson, C.J. 2009. Introducing mothur: open-source, platform-independent, community-supported software for describing and comparing microbial communities. *Applied and Environmental Microbiology* **75**: 7537–7541.

Wang, Q., Garrity, G.M., Tiedje, J.M., and Cole, J.R. 2007. Naive Bayesian classifier for rapid assignment of rRNA sequences into the new bacterial taxonomy. *Applied and Environmental Microbiology* **73**: 5261–5267.

Waterhouse, A.M., Procter, J.B., Martin, D.M.A., Clamp, M., and Barton, G.J. 2009. Jalview Version 2—a multiple sequence alignment editor and analysis workbench. *Bioinformatics*  **25** (9): 1189–1191.

Wiklund, H., Taylor, J.D., Dahlgren, T.G., Todt, C., Ikebe, C., Rabone, M., and Glover, A.G. 2017. Abyssal fauna of the UK-1 polymetallic nodule exploration area, Clarion-Clipperton Zone, central Pacific Ocean: Mollusca. *ZooKeys* (707): 1–46.

Zhang, J., Kobert, K., Flouri, T., and Stamatakis, A. 2014. PEAR: a fast and accurate Illumina Paired-End reAd mergeR. *Bioinformatics*  **30** (5): 614–620.
