# Supplementary material for: Larval assemblages over the abyssal plain in the Pacific are highly diverse and spatially patchy
Source: PeerJ. 2019 Sep 26;7:e7691. doi: 10.7717/peerj.7691 (PMC6766376; doi:10.7717/peerj.7691)
Supplement: Supplemental Information 1 [file peerj-07-7691-s013.docx]

**Supplementary File 2 for:**

**Larval assemblages over the abyssal plain in the Pacific are highly diverse and spatially patchy**

Oliver Kersten, Eric W. Vetter, Michelle J. Jungbluth, Craig R. Smith, Erica Goetze

**Table References:**

1. Rouse, G. W., Goffredi, S. K. and Vrijenhoek, R. C. 2004. Osedax: bone-eating marine worms with dwarf males. *Science* **305**: 668–671.

2. Boxshall, G. A. and Halsey, S. H. 2004. *An introduction to copepod diversity*. Ray Society.

3. Vincx, M. *et al.* 1994. Meiobenthos of the Deep Northeast Atlantic. in *Advances in Marine Biology* (eds. Blaxter, J. H. S. & Southward, A. J.) **30**: 1–88. Academic Press.

4. Kirkegaard, J. B. 1996. Bathyal and Abyssal Polychaetes (Sedentary Species I). *Galathea Report* **17**: 57-77.

5. Silva, C. F. *et al.* 2016. A new Capitella polychaete worm (Annelida: Capitellidae) living inside whale bones in the abyssal South Atlantic. *Deep Sea Research Part I* **108**: 23–31.

6. OBIS. 2018. Ocean Biogeographic Information System. *Intergovernmental Oceanographic Commission of UNESCO*.

7. Méndez, N. 2007. Relationships between deep-water polychaete fauna and environmental factors in the southeastern Gulf of California, Mexico. *Scientia Marina* **71**: 605–622.

8. Nishi, E. & Rouse, G. W. 2007. A new species of Phyllochaetopterus (Chaetopteridae: Annelida) from near hydrothermal vents in the Lau Basin, western Pacific Ocean. *Zootaxa* **1621**: 55–64.

9. De Smet, B. *et al.* 2017. The Community Structure of Deep-Sea Macrofauna Associated with Polymetallic Nodules in the Eastern Part of the Clarion-Clipperton Fracture Zone. *Frontiers in Marine Science* **4**: 103.

10. Arbizu, P. M. 2006. Phylogenetic relationships within Schminkepinellidae fam. n., a new monophyletic group of marine cyclopinids (Cyclopoida: Copepoda), description of two new genera and four new species. *Invertebrate Zoology* **3**: 185–207.

11. Humes, A. G. 1999. Copepoda (Cyclopinidae and Misophriidae) from a deep-sea hydrothermal site in the northeastern Pacific. *Journal of Natural History* **33**: 961–978.

12. Chertoprud, E., Abramova, E., Korsun, S., Martynov, F. and Garlitska, L. 2018. Composition of Harpacticoida (Crustacea, Copepoda) of the Laptev Sea in comparison with faunas of adjacent Arctic seas. *Polar Biology* **41**: 697–712.

13. Cho, D. H., Wi, J. H. and Suh, H.-L. 2016. Two new species of the deep-sea genus Parameiropsis (Copepoda: Harpacticoida) from the eastern central Pacific. *Zootaxa* **4132**: 521–539.

14. Amon, D. J. *et al.* 2017. Megafauna of the UKSRL exploration contract area and eastern Clarion-Clipperton Zone in the Pacific Ocean: Echinodermata. *Biodiversity Data Journal* **5**: e11794.

15. Sirenko, B. 2008. Bathyal chitons (Mollusca, Polyplacophora) from off New Caledonia and Vanuatu: families Callochitonidae, Ischnochitonidae and Loricidae. *Mémoires du Muséum national d’Histoire naturelle (1993)*.

16. Lanterbecq, D., Rouse, G. W., Milinkovitch, M. C. and Eeckhaut, I. 2006. Molecular phylogenetic analyses indicate multiple independent emergences of parasitism in Myzostomida (Protostomia). *Systematic Biology* **55**: 208–227.

17. Lanterbecq, D., Rouse, G. W. and Eeckhaut, I. 2010. Evidence for cospeciation events in the host–symbiont system involving crinoids (Echinodermata) and their obligate associates, the myzostomids (Myzostomida, Annelida). *Molecular Phylogenetic Evolution* **54**: 357–37.

18. Worsaae, K. and Kristensen, R. M. 2003. A new species of Paranerilla (Polychaeta: Nerillidae) from northeast Greenland waters, arctic ocean. *Cahiers de Biologie Marine***44**(1): 23-39.

19. Worsaae, K., Sterrer, W. and Iliffe, T. M. 2004. *Longipalpa saltatrix*, a new genus and species of the meiofaunal family Nerillidae (Annelida: Polychaeta) from an anchihaline cave in Bermuda. *Proceedings of the Biologcal Society of Washington* 346–362.

20. Schüller, M. and Ebbe, B. 2007. Global distributional patterns of selected deep-sea Polychaeta (Annelida) from the Southern Ocean. *Deep Sea Research Part 2 Topical Studies in Oceanography* **54**: 1737–1751.

21. García-Garza, M. E. and León-González, J. A. D. 2011. Review of the Capitellidae (Annelida, Polychaeta) from the Eastern Tropical Pacific region, with notes on selected species. *Zookeys* **151**: 17–52.

22. Hartman, O. and Fauchald, K. 1971. Deep-water benthic polychaetous annelids off New England to Bermuda and other North Atlantic areas. Part II. *Allan Hancock Monographs in Marine Biology* **6**: 1-327.

23. Taboada, S. *et al.* 2013. Two new Antarctic Ophryotrocha (Annelida: Dorvilleidae) described from shallow-water whale bones. *Polar Biology* **36**: 1031–1045.

24. Amon, D. J., Hilario, A., Arbizu, P. M. and Smith, C. R. 2017. Observations of organic falls from the abyssal Clarion-Clipperton Zone in the tropical eastern Pacific Ocean. *Marine Biodiversity* **47**: 311–321.

25. Neal, L., Barnich, R., Wiklund, H. and Glover, A. G. 2012. A new genus and species of Polynoidae (Annelida, Polychaeta) from Pine Island Bay, Amundsen Sea, Southern Ocean-a region of high taxonomic novelty. *Zootaxa* **3542** (1): 80–88.

26. Neal, L., Brasier, M. J. and Wiklund, H. 2018. Six new species of Macellicephala (Annelida: Polynoidae) from the Southern Ocean and south Atlantic with re-description of type species. *Zootaxa* **4455**: 1–34.

27. Amon, D. J. *et al.* 2017. Megafauna of the UKSRL exploration contract area and eastern Clarion-Clipperton Zone in the Pacific Ocean: Annelida, Arthropoda, Bryozoa, Chordata, Ctenophora, Mollusca. *Biodivers Data Journal* **5**: e14598.

28. Kupriyanova, E. K. *et al.* 2014. Serpulids living deep: calcareous tubeworms beyond the abyss. *Deep Sea Research Part I* **90**: 91–104.

29. Galkin, S. V. and Goroslavskaya, E. I. 2008. Bottom fauna associated with mussel beds and alvinellid communities in the hydrothermal field at 9° N of the East Pacific Rise. *Oceanology* **48**: 509–516.

30. Fauchald, K. and Hancock, D. R. 1981. Deep-water polychaetes from a transect off central Oregon. *Allan Hancock Monographs in Marine Biology* **11**: 1-73.

31. Meißner, K., Bick, A., Guggolz, T. and Götting, M. 2014. Spionidae (Polychaeta: Canalipalpata: Spionida) from seamounts in the NE Atlantic. *Zootaxa* **3786**: 201–245.

32. Jirkov, I. A., Ravara, A. and Cunha, M. R. 2018. *Amphitrite fauveli sp.n*. (Polychaeta: Terebellidae) from the Bay of Biscay and the Gulf of Cadiz (NE Atlantic). *Invertebrate Zoology* **15**: 85–91.

33. Petersen, G. H. *et al.* 2996. Red List of macrofaunal benthic invertebrates of the Wadden Sea. *Helgoländer Meeresuntersuchungen* **50**: 69–76.

34. Araya, J. F. 2016. New records of deep-sea sea spiders (Chelicerata: Pycnogonida) in the southeastern Pacific. *Marine Biodiversity* **46**: 725–729.

35. WoRMS Editorial Board. 2019. World Register of Marine Species. Available from http://www.marinespecies.org at VLIZ. Accessed 12-2018. doi:10.14284/170

36. Wyngaard, G. A., Rocha, C. and Pepato, A. 2011. Familial level phylogeny of free-living cyclopoids (Copepoda), inferred from partial 18S ribosomal DNA. *Studies on Freshwater Copepoda: A Volume in Honour of Bernard Dussart.* 507-544. Brill Publishing.

37. Arbizu, P. M. 1997. A new genus of cyclopinid copepods (Crustacea), with a redescription of *Smirnovipina barentsiana comb. nov.* (Smirnov, 1931). *Sarsia* **82**: 313–323.

38. Seifried, S. and Martínez Arbizu, P. 2008. A new and exceptional species of *Bradya* Boeck, 1873 (Copepoda: Harpacticoida: Ectinosomatidae) from the abyssal plain of the Angola Basin and the variability of deep-sea Harpacticoida. *Zootaxa* **1866**: 303–322.

39. Seifried, S., Plum, C. and Schulz, M. 2007. A new species of *Parabradya* Lang, 1944 (Copepoda: Harpacticoida: Ectinosomatidae) from the abyssal plain of the Angola Basin. *Zootaxa* **1432**: 1–21.

40. George, K. H. and Wiest, J. 2015. *Pseudometeorina mystica gen. et sp. nov*., a new Idyanthidae Lang incertae sedis (Copepoda, Harpacticoida) from the Guinea Basin (eastern tropical Atlantic). *Marine Biodiversity* **45**: 569–580.

41. Gollner, S., Ivanenko, V. N., Arbizu, P. M. and Bright, M. 2010. Advances in taxonomy, ecology, and biogeography of Dirivultidae (copepoda) associated with chemosynthetic environments in the deep sea. *PLoS One* **5**. e9801.

42. Mangena, T., Jordaan, B. P. and Dippenaar, S. M. 2014. Phylogenetic relationships and genetic diversity of *Nemesis* (Risso, 1826) species found on different elasmobranch host species off the KwaZulu-Natal coast, South Africa. *African Journal of Marine Science* **36**: 163–173.

43. Shalaeva, K. 2014. An illustrated catalogue of the scalpellid barnacles (Crustacea: Cirripedia: Scalpellidae) collected during the HMS ‘Challenger’ expedition and deposited in the Natural History Museum, London. *Zootaxa* **3804**: 1–63.

44. Ingram, C. L. and Hessler, R. R. 1983. Distribution and behavior of scavenging amphipods from the central North Pacific. *Deep Sea Research A* **30**: 683–706.

45. Martin, J. W. and Pettit, G. 1998. *Caprella bathytatos* new species (Crustacea, Amphipoda, Caprellidae), from the mouthparts of the crab *Macroregonia macrochira* Sakai (Brachyura, Majidae) in the vicinity of deep-sea hydrothermal vents off British Columbia. *Bulletin of Marine Science* **63**: 189–198.

46. Takeuchi, I., Tomikawa, K. and Lindsay, D. 2016. A new genus and species of Phtisicidae (Crustacea: Amphipoda) from abyssal depths in the Japan Trench, with special reference to similarities with Southern Ocean genera. *Journal of Crustacean Biology* **36**: 495–506.

47. Verdi, A. and Celentano, E. 2008. New host and distribution records of *Caprella bathytatos* Martin & Pettit, 1998 (Amphipoda, Caprellidae). *Crustaceana* **81**: 673–678.

48. Lowry, J. K. and Kilgallen, N. M. 2014. A generic review of the Lysianassoid family Uristidae and descriptions of new taxa from Australian waters (Crustacea, Amphipoda, Uristidae). *Zootaxa* **3867**: 1–92.

49. Lacey, N. C. *et al.* 2016. Community structure and diversity of scavenging amphipods from bathyal to hadal depths in three South Pacific Trenches. *Deep Sea Research Part I* **111**: 121–137.

50. Thurston, M. H. 1990. Abyssal necrophagous amphipods (Crustacea: Amphipoda) in the northeast and tropical Atlantic Ocean. *Progress in Oceanography* **24**: 257–274.

51. Lowry, J. K. and De Broyer, C. 2008. Alicellidae and Valettiopsidae, two new callynophorate families (Crustacea: Amphipoda). *Zootaxa* **1843**: 57–66.

52. Schmalfuss, H. 2003. World catalog of terrestrial isopods (Isopoda: Oniscidea). *Stuttgarter Beiträge zur Naturkunde, Serie A*, **654**: 341 pp. Stuttgart.

53. Janssen, A. *et al.* 2015. A reverse taxonomic approach to assess macrofaunal distribution patterns in abyssal Pacific polymetallic nodule fields. *PLoS One* **10**: e0117790.

54. Malyutina, M. and Brandt, A. 2007. Diversity and zoogeography of Antarctic deep-sea Munnopsidae (Crustacea, Isopoda, Asellota). *Deep Sea Research Part 2 Topical Studies in Oceanography* **54**: 1790–1805.

55. Osborn, K. J. 2009. Relationships within the Munnopsidae (Crustacea, Isopoda, Asellota) based on three genes. *Zoologica Scripta* **38**: 617–635.

56. Knudsen, S. W., Kirkegaard, M. and Olesen, J. 2009. The tantulocarid genus *Arcticotantalus* removed from Basipodellidae into Deoterthridae (Crustacea: Maxillopoda) after the description of a new species from Greenland, with first live photographs and an overview of the class. *Zootaxa* **2035**: 41–68.

57. Kolbasov, G. A., Yu. Sinev, A. and Tchesunov, A. V. 2008. External morphology of *Arcticotantulus* pertzovi (Tantulocarida, Basipodellidae), a microscopic crustacean parasite from the White Sea. *Entomological Review* **88** (9): 1192–1207.

58. Massard, J. A. and Geimer, G. 2008. Global diversity of bryozoans (Bryozoa or Ectoprocta) in freshwater: an update. *Bulletin de la Société des naturalistes luxembourgeois* **109**, 139–148.

59. Grischenko, A. V. and Chernyshev, A. V. 2015. *Triticella minini*--a new ctenostome bryozoan from the abyssal plain adjacent to the Kuril-Kamchatka Trench. *Deep Sea Research Part 2 Topical Studies in Oceanography* **111**: 343–350.

60. O’Loughlin, P. M., Waters, J. M. and Roy, M. S. 2002. Description of a new species of *Patiriella* from New Zealand, and review of *Patiriella regularis* (Echinodermata, Asteroidea) based on morphological and molecular data. *Journal of the Royal Society of New Zealand* **32**: 697–711.

61. Hunter, R. L. and Halanych, K. M. 2010. Phylogeography of the Antarctic planktotrophic brittle star *Ophionotus victoriae* reveals genetic structure inconsistent with early life history. *Marine Biology* **157**: 1693–1704.

62. Smirnov, I. S., Piepenburg, D., Ahearn, C. and Juterzenka, K. von. 2014. Deep-sea fauna of European seas: an annotated species check-list of benthic invertebrates living deeper than 2000 m in the seas bordering Europe: Ophiuroidea. *Invertebrate Zoology* **11**: 192–209.

63. Mironov, A. N. 2006. Echinoids from seamounts of the north-eastern Atlantic; onshore/offshore gradients in species distribution. in *Biogeography of the North Atlantic Seamounts* (eds. Mironov et al.). pp. 96-133. KMK Scientific Press, Russian Academy of Sciences, P.P. Shirshov Institute of Oceanology, Moscow.

64. Zigler, K. S. and Lessios, H. A. 2004. Speciation on the coasts of the new world: phylogeography and the evolution of bindin in the sea urchin genus *Lytechinus*. *Evolution* **58**: 1225–1241.

65. Borisanova, A. O., Chernyshev, A. V., Neretina, T. V. and Stupnikova, A. N. 2015. Description and phylogenetic position of the first abyssal solitary kamptozoan species from the Kuril-Kamchatka Trench area: *Loxosomella profundorum sp. nov.* (Kamptozoa: Loxosomatidae). *Deep Sea Research Part 2 Topical Studies in Oceanography* **111**: 351–356.

66. Sasaki, T., Warén, A., Kano, Y., Okutani, T. and Fujikura, K. 2010. Gastropods from Recent Hot Vents and Cold Seeps: Systematics, Diversity and Life Strategies. in *The Vent and Seep Biota: Aspects from Microbes to Ecosystems* (ed. Kiel, S.): 169–254. Springer Netherlands.

67. Marshall, B. A. 1991. Mollusca Gastropoda: Seguenziidae from New Caledonia and the Loyalty Islands. In *Résultats des Campagnes MUSORSTOM 7. Mémoires du Muséum national d'Histoire naturelle. Série A, Zoologie* (Eds. Crosnier, A. et al.) **150**: 41–109. Éditions du Muséum: Paris.

68. Bik, H. M., Thomas, W. K., Lunt, D. H. and Lambshead, P. J. D. 2010. Low endemism, continued deep-shallow interchanges, and evidence for cosmopolitan distributions in free-living marine nematodes (Order Enoplida). *BMC Evolutionary Biology* **10**: 389.

69. Lörz, A. N. *et al.* 2012. A review of deep-sea benthic biodiversity associated with trench, canyon and abyssal habitats below 1500 m depth in New Zealand waters. *New Zealand Aquatic Environment and Biodiversity Report* **92**: 1–159.

70. Saiz, J. I., Bustamante, M. and Tajadura, J. 2018. A census of deep-water sipunculans (Sipuncula). *Marine Biodiversity* **48**: 449–464.

71. Hooge, M. D. and Tyler, S. 2003. Two new acoels (Acoela, Platyhelminthes) from the central coast of California. *Zootaxa* **131**: 1–14.

72. Hasegawa, K. 1997. Sunken Wood-Associated Gastropods Collected from Suruga Bay, Pacific Side of the Central Honshu, Japan, with Descriptions of 12 New Species. *National Science Museum Monographs* **12**: 59–123.

73. Kunze, T., Heß, M., Beck, F. and Haszprunar, G. 2008. Skeneimorph gastropods in Neomphalina and Vetigastropoda—A preliminary report. *Zoosymposia* **1**: 119–131.

74. Waren, A. and Bouchet, P. 1993. New records, species, genera, and a new family of gastropods from hydrothermal vents and hydrocarbon seeps. *Zoologica Scripta* **22**: 1–90.

75. McLean, J. H. 1970. Notes on the deep water Calliostomas of the Panamic Province with descriptions of six new species. *Veliger* **12**: 421–426.

76. McClain, C. R. and Lundsten, L. 2015. Assemblage structure is related to slope and depth on a deep offshore Pacific seamount chain. *Marine Ecology* **36**: 210–220.

77. Dijkstra, H. H. and Marshall, B. A. 2008. The Recent Pectinoidea of the New Zealand region (Mollusca: Bivalvia: Propeamussiidae, Pectinidae and Spondylidae. *Molluscan Research* **28**: 1-88.

78. Kamenev, G. M. 2018. Four new species of the family Propeamussiidae (Mollusca: Bivalvia) from the abyssal zone of the northwestern Pacific, with notes on *Catillopecten squamiformis* (Bernard, 1978). *Marine Biodiversity* **48**: 647–676.

79. Wiklund, H. *et al.* 2017. Abyssal fauna of the UK-1 polymetallic nodule exploration area, Clarion-Clipperton Zone, central Pacific Ocean: Mollusca. *Zookeys* **707**: 1–46.

80. Voight, J. R. 2015. Xylotrophic bivalves: aspects of their biology and the impacts of humans. *Journal of Molluscan Studies* **81**: 175–186.

81. Dahlgren, T. G. and Pleijel, F. 1995. On the generic allocation of *Chrysopetalum caecum* Langerhans, 1880 (Polychaeta, Chrysopetalidae). *Mitteilungen aus dem Hamburgischen Zoologischen Museum und Institut, Ergänzungsband* **92**: 159–173.

82. Watson, C., Chivers, A. J., Narayanaswamy, B. E., Lamont, P. and Turnewitsch, R. 2014. Chrysopetalidae (Annelida: Phyllodocida) from the Senghor Seamount, north-east Atlantic: taxa with deep-sea affinities and morphological adaptations. *Memoirs of Museum Victoria* **71**: 311-325.

83. Levin, L. A., Mendoza, G. F., Konotchick, T. and Lee, R. 2009. Macrobenthos community structure and trophic relationships within active and inactive Pacific hydrothermal sediments. *Deep Sea Research Part 2 Topical Studies in Oceanography* **56**: 1632–1648.

84. Summers, M., Pleijel, F. and Rouse, G. W. 2015. Whale falls, multiple colonisations of the deep, and the phylogeny of Hesionidae (Annelida). *Invertebrate Systematics* **29**: 105–123.

85. Alfaro-Lucas, J. M., Shimabukuro, M., Ogata, I. V., Fujiwara, Y. and Sumida, P. Y. G. 2018. Trophic structure and chemosynthesis contributions to heterotrophic fauna inhabiting an abyssal whale carcass. *Marine Ecology Progress Series* **596**: 1–12.

86. Levenstein, R. Y. 1972. Ecology and Zoogeography of some Polychaeta Representatives of the Abyssal Pacific. *Proceedings of the Royal Society of Edinburgh, Section B: Biological Sciences* **73**: 171–181.

87. Guidi-Guilvard, L. D., Thistle, D., Khripounoff, A. and Gasparini, S. 2009. Dynamics of benthic copepods and other meiofauna in the benthic boundary layer of the deep NW Mediterranean Sea. *Marine Ecology Progress Series* **396**: 181–195.

88. Schmidt, C., Lins, L. and Brandt, A. 2018. Harpacticoida (Crustacea, Copepoda) across a longitudinal transect of the Vema Fracture Zone and along a depth gradient in the Puerto Rico trench. *Deep Sea Research Part 2 Topical Studies in Oceanography* **148**: 236–250.

89. Shulenberger, E. and Laurens Barnard, J. 1976. Amphipods from an Abyssal Trap Set in the North Pacific Gyre. *Crustaceana* **31**: 241–258.

90. Fonseca, V. G. *et al.* 2010. Second-generation environmental sequencing unmasks marine metazoan biodiversity. *Nature Communications* **1**: 98.

91. Machida, R. J. and Knowlton, N. 2012. PCR primers for metazoan nuclear 18S and 28S ribosomal DNA sequences. *PLoS One* **7**: e46180.

92. Leray, M. *et al.* 2013. A new versatile primer set targeting a short fragment of the mitochondrial COI region for metabarcoding metazoan diversity: application for characterizing coral reef fish gut contents. *Frontiers in Zoology* **10**: 34.

93. Folmer, O., Black, M., Hoeh, W., Lutz, R., and Vrijenhoek, R. 1994. DNA primers for amplification of mitochondrial cytochrome c oxidase subunit I from diverse metazoan invertebrates. *Molecular Marine Biology and Biotechnology* **3**: 294–299.

94. Carr, C. M., Hardy, S. M., Brown, T. M., Macdonald, T. A. and Hebert, P. D. N. 2011. A tri-oceanic perspective: DNA barcoding reveals geographic structure and cryptic diversity in Canadian polychaetes. *PLoS One* **6**: e22232.

95. Nygren, A. and Sundberg, P. 2003. Phylogeny and evolution of reproductive modes in Autolytinae (Syllidae, Annelida). *Molecular Phylogenetics and Evolution* **29**: 235–249.

96. Cohen, B. L., Gawthrop, A. and Cavalier–Smith, T. 1998. Molecular phylogeny of brachiopods and phoronids based on nuclear–encoded small subunit ribosomal RNA gene sequences. *Philosophical Transactions of the Royal Society London B: Biological Sciences* **353**: 2039–2061.

97. Medlin, L., Elwood, H. J., Stickel, S. and Sogin, M. L. 1988. The characterization of enzymatically amplified eukaryotic 16S-like rRNA-coding regions. *Gene* **71**: 491–499.

98. Wilson, G.D.F. 2017. Macrofauna abundance, species diversity and turnover at three sites in the Clipperton-Clarion Fracture Zone. *Marine Biodiversity* **47** (2): 323–347.
